# Generation and validation of a conditional knockout mouse model for the study of the Smith-Lemli-Opitz syndrome

**DOI:** 10.1194/jlr.RA120001101

**Published:** 2020-11-22

**Authors:** Babunageswararao Kanuri, Vincent Fong, Sithara Raju Ponny, Keri A. Tallman, Sriganesh Ramachandra Rao, Ned Porter, Steven J. Fliesler, Shailendra B. Patel

**Affiliations:** 1 Division of Endocrinology, Diabetes and Metabolism, University of Cincinnati, Cincinnati, OH, USA; 2 Division of Human Genetics, Cincinnati Children's Hospital Medical Center, Cincinnati, OH, USA; 3 Department of Chemistry and Vanderbilt Institute of Chemical Biology, Vanderbilt University, Nashville, TN, USA; 4 Departments of Ophthalmology and Biochemistry, Jacobs School of Medicine and Biomedical Sciences, University at Buffalo-State University of New York, Buffalo, NY, USA; 5 Graduate Program in Neuroscience, University at Buffalo- State University of New York, Buffalo, NY, USA; 6 Research Service, VA Western New York Healthcare System, Buffalo, NY, USA

**Keywords:** dysmorphology, cholesterol synthesis, 7-dehydrocholesterol, Smith-Lemli-Opitz syndrome, ABCA1, ATP binding cassette subfamily A member 1, ABCB11, ATP binding cassette subfamily B member 11, ABCG1/5/8, ATP binding cassette subfamily G member 1/5/8, APO, apolipoprotein, BHT, 2,6-di-*tert*-butyl-4-methylphenol, CD36, cluster of differentiation 36, CEACAM1, carcinoembryonic antigen-related cell adhesion molecule 1, CHCl_3_, trichloromethane, Chol, cholesterol, CYP7A1, cytochrome P450 family 7 subfamily A member 1, CYP27A1, cytochrome P450 family 27 subfamily A member 1, CYP8B1, cytochrome P450 family 8 subfamily B member 1, Des, desmosterol, 7-DHC, 7-dehydrocholesterol, 8-DHC, 8-dehydrocholesterol, DHCR24, 3β-hydroxysterol-Δ24 reductase, DHCR7, 3β-hydroxysterol-Δ7 reductase, 7-DHD, 7-dehydrodesmosterol, DHL, 24-dehydrolathosterol, dHLan, 24-dihydrolanosterol, DMAP, 4-dimethylaminopyridine, DMG, dimethylglycine, 14d-Zym, 14-dehydrozymosterol, 14d-Zyme, 14-dehydrozymostenol, ES, embryonic stem, ESI, electrospray ionization, Et_3_N, triethylamine, FAS, fatty acid synthase, FDFT1, Farnesyl-diphosphate farnesyl transferase 1, GEO, Gene Expression Omnibus, GTT, glucose tolerance test, HMGCR, 3-hydroxy-3-methylglutaryl-CoA reductase, IACUC, institutional animal care and use committee, ITT, insulin tolerance test, Lan, lanosterol, Lath, lathosterol, LC-MS, liquid chromatography-mass spectrometry, LDLR, low density lipoprotein receptor, LIPC, hepatic triacylglycerol lipase, LKO, liver-specific knockout, LXR, liver X receptor, MTTP, microsomal triglyceride transfer protein, NR1H2/3/4/5, nuclear receptor subfamily 1 group H member 2/3/4/5, PCSK9, proprotein convertase subtilisin/kexin type 9, PPh_3_, triphenylphosphine, PPARA, peroxisome proliferator activated receptor alpha, PPARG, peroxisome proliferator activated receptor gamma, SCARB1, scavenger receptor class B member 1, SREBF1/2, sterol regulatory element binding transcription factor half, SLOS, Smith-Lemli-Opitz syndrome, SRM, selected reaction monitoring, TPM, transcript per million, UPLC, ultra performance liquid chromatography, Zyme, zymostenol, Zym, zymosterol

## Abstract

Smith-Lemli-Opitz Syndrome (SLOS) is a developmental disorder (OMIM #270400) caused by autosomal recessive mutations in the *Dhcr7* gene, which encodes the enzyme 3β-hydroxysterol-Δ7 reductase. SLOS patients present clinically with dysmorphology and neurological, behavioral, and cognitive defects, with characteristically elevated levels of 7-dehydrocholesterol (7-DHC) in all bodily tissues and fluids. Previous mouse models of SLOS have been hampered by postnatal lethality when *Dhcr7* is knocked out globally, while a hypomorphic mouse model showed improvement in the biochemical phenotype with aging and did not manifest most other characteristic features of SLOS. We report the generation of a conditional knockout of *Dhcr7* (*Dhcr7*^flx/flx^), validated by generating a mouse with a liver-specific deletion (*Dhcr7*^L-KO^). Phenotypic characterization of liver-specific knockout mice revealed no significant changes in viability, fertility, growth curves, liver architecture, hepatic triglyceride secretion, or parameters of systemic glucose homeostasis. Furthermore, qPCR and RNA-Seq analyses of livers revealed no perturbations in pathways responsible for cholesterol synthesis, either in male or in female *Dhcr7*^*L-KO*^ mice, suggesting that hepatic disruption of postsqualene cholesterol synthesis leads to minimal impact on sterol metabolism in the liver. This validated conditional Dhcr7 knockout model may now allow us to systematically explore the pathophysiology of SLOS, by allowing for temporal, cell and tissue-specific loss of DHCR7.

The enzyme 3β-hydroxysterol-Δ7 reductase (DHCR7; EC 1.3.1.21) catalyzes the reduction of the C7-8 double bond in 7-dehydrosterol precursors in both the Bloch and Kandutsch-Russell (K-R) pathways (1, 2) necessary for the synthesis of cholesterol. This results in the conversion of 7-dehydrodesmosterol (7-DHD) to desmosterol and 7-dehydrocholesterol (7-DHC) to cholesterol. Desmosterol is converted to cholesterol in the subsequent step of the Bloch pathway by 3β-hydroxysterol-Δ24 reductase (DHCR24; EC 1.3.1.72) (1, 2). Located on chromosome 7, loss-of-function homozygous or compound heterozygous mutations in *DHCR7* result in the Smith-Lemli-Opitz-Syndrome (SLOS, OMIM #270400) (3, 4, 5), which is the most common cause of inborn errors of cholesterol synthesis (6, 7). Although SLOS has been reported worldwide, it seems more prevalent in the Caucasian population, with an overall carrier frequency of mutant *DHCR7* alleles of about 1.4%, but with a prevalence of 1:20,000 to 1:60,000 live births (8, 9). While there is impairment of endogenous cholesterol synthesis, maternal-fetal cholesterol transfer in utero and postnatal dietary cholesterol both provide partial cholesterol replenishment in these patients (10) and may lead to some mitigation of the phenotype. In addition to its distinctive biochemical phenotype of elevated plasma and tissue 7-DHC (and its spontaneous isomer 8-dehydrocholesterol, 8-DHC) (4, 5, 6), SLOS patients exhibit multiple abnormalities, which include craniofacial and midline malformations, neurodevelopmental and cognitive deficits (including autism spectrum disorder), ambiguous genitalia, and 2–3 syndactyly as more frequent manifestations (3, 6).

Initial attempts at generation of a viable mouse model of SLOS by deleting *Dhcr7* exons 3–5 or exon 9 met with limited success as no knockout pups survived beyond postnatal day 1 (11, 12). Morphometric and histological analysis of these pups showed immature lungs, enlarged bladders, and cleft palates (11, 12). Subsequent studies utilized site-directed mutagenesis to reproduce the most common missense SLOS mutation, T93M, and generated homozygous (*Dhcr7*^*T93M/T93M*^) and compound heterozygous (*Dhcr7*^*T93M/Δ3-5*^) mice (13, 14). *Dhcr7*^*T93M/Δ3-5*^ mice demonstrated some phenotypic changes of SLOS, including CNS ventricular dilatation and 2–3 syndactyly (14, 15). Both of these mice strains were viable and had elevated 7-DHC levels in tissues. However, the biochemical defect improved with age (13, 14), suggesting that residual enzymatic activity and/or compensatory mechanism(s) resulted in significant amelioration of the biochemical defect. In contrast, the SLOS phenotype in humans does not reverse or improve with age (11, 12), thus limiting the utility of this model. An alternative to genetic models is the use of specific pharmacological inhibitors of DHCR7, such as AY9944 and BM15.766, to induce a SLOS phenotype (16, 17, 18, 19). However, tissue-specific differences in drug bioavailability and cholesterol turnover rates limit the ability to accurately account for tissue enzyme inhibition; in addition, off-target effects of these pharmacological agents cannot be discounted.

In the current study, we sought to develop a genetic mouse model with consistent and stable deletion of *Dhcr7* that would survive into adulthood and also allow detailed studies of specific tissues. We generated a conditional knockout mouse strain by creating a floxed mouse with LoxP sites flanking exon 8 of *Dhcr7*, which would allow for complete deletion of *Dhcr7* in specific tissues and cell types or under specific conditions with a Cre recombinase. The system was validated by generating a mouse strain with liver-specific deletion of *Dhcr7* (*Dhcr7*^flx/flx,^Alb-Cre^+^), referred to as *Dhcr7*^L-KO^, using a Cre recombinase directed by the albumin promoter.

## Materials and methods

### Chemical reagents and solvents

Unless otherwise noted, all chemicals were purchased from Sigma-Aldrich (St. Louis, MO). HPLC-grade solvents were purchased from Thermo Fisher Scientific Inc. (Waltham, MA). All deuterated sterol standards are available from Kerafast, Inc. (Boston, MA). However, a manuscript describing these standards will be published separately (Tallman, K.A. and Porter, N. A., manuscript in preparation).

### Animal care

All animal protocols were approved by the University of Cincinnati IACUC, Cincinnati, OH. All mice used in the study were purchased from Jackson Labs (Bar Harbor, ME); Alb-Cre [B6.Cg-*Speer6-ps1*^*Tg(Alb-cre)21Mgn*^/J; #003574], flp recombinase [B6.129S4-*Gt(ROSA)26Sor*^*tm1(FLP1)Dym*^/RainJ; #009086] and ER-Cre [B6.Cg-*Ndor1*^*Tg(UBC-cre/ERT2)1Ejb*^/2J Stock; #008085], as well as stock C57Bl/6J. Mice were group-housed in individually ventilated PIV cages maintained on 12 h:12 h light and dark cycles and fed a standard chow diet (Envigo #7912; Harlan Teklad, Madison, WI) with access to water ad libitum.

### Generation of conditional Dhcr7 knockout mice

A fee-based commercial service (Cyagen Biosciences Inc., Santa Clara, CA) was used to make the conditional mouse, employing ES cell-mediated gene targeting. In brief, a targeting vector was designed with a Neo cassette, flanked by Frt sites, and *Dhcr7* Exon 8, flanked by LoxP sites, and was introduced into Cyagen's proprietary TurboKnockout ES cells (on a C57Bl/6N background) by electroporation (20). The targeted ES cells were introduced into host embryos, and 4–8 microinjected cell embryos were then surgically transferred into pseudo-pregnant (surrogate) mothers to generate F0 heterozygous floxed mice (*Dhcr7*^flx/wt^) on a C57Bl/6N background, then bred to a C57Bl/6J background, and screened against the inheritance of the *rd8* mutant allele (21) from the C57Bl/6N background in the first round. Liver-specific knockout (LKO) mice were generated using Cre-LoxP recombinase technology (22). *Dhcr7*^flx/wt^ mice were first crossed with Alb-Cre (23), and the resultant heterozygotes positive for Cre recombinase (*Dhcr7*^flx/wt,^ Alb-Cre+) were bred with mice carrying flp recombinase (24) to remove Neo cassette (as the first crosses showed this cassette led to a null allele). Brother and sister matings of the Neo deleted heterozygotes finally generated liver-specific *Dhcr7* knockout (*Dhcr7*^L-KO^) mice. Subsequently, backcrossing to C57Bl/6J mice for at least six generations (N = 6) created genetically clean congenic floxed *Dhcr7*^*L-KO*^ mice. All the mice genotypes were tested by tail snip PCR for the presence of the floxed allele and Cre recombinase. The list of primers used for detection of deletion of floxed allele, Cre recombinase, RNA exon splicing (exon 5–9, exon 6–9, exon 7–9), and genomic DNA exon 8 deletion are presented in supplemental Table S1. A subset of *Dhcr7*^flx/wt^ was mated with Estrogen receptor-Cre mice (25) to generate mice bearing both the ER-Cre allele and the floxed allele. Mice were injected daily for 3 days with 75 mg/kg tamoxifen (T5648; Sigma-Aldrich, St. Louis, MO) dissolved in canola oil (10 mg/ml) to globally delete exon 8 and then bred together after 8 weeks to obtain germline transmission of the deleted exon 8 (*Dhcr7*^Δ8/+^).

### Blood and tissue collection and histological staining

Heparinized submandibular blood samples were drawn weekly (7–12 weeks of age, after 4 h of fasting) for time course experiments. The start times of fasting were between 8 and 10 AM and staggered to minimize the variability from first to last animal. The isolated blood was centrifuged at 2,500 rpm (664 *g*) for 15 min at 4°C, and the resulting plasma fraction was collected and stored at –80°C. For bile collection, mice were fasted for 4 h and then euthanized by CO_2_ inhalation and cervical dislocation, an incision was made below the diaphragm to initially reveal the liver and gall bladder followed by removal of the gall bladder. With a gentle puncture using a 25G needle, bile from the gall bladder was drained into 1.5-ml microcentrifuge tubes and stored at –80°C along with plasma samples until further use.

Terminal tissue collection experiments were performed using mice 12–14 weeks of age after euthanizing with CO_2_ inhalation. Following harvest, the liver and brain tissues were immediately flash frozen in liquid N_2_ or collected in RNA*later* (AM7021; Ambion, Austin, TX) and stored at –80°C for subsequent quantitation of tissue sterols or RNA isolations (26). A small portion of the liver was rinsed in ice-cold phosphate-buffered saline (PBS; pH 7.4), fixed in 10% neutral buffered formalin (5725; Fisher Scientific, Pittsburg, PA), and embedded in paraffin using routine procedures (26). Histological tissue sections (ca, 5-μm thickness each) were obtained by conventional microtomy, collected onto glass microscope slides, deparaffinized through graded concentrations of ethanol, and then stained with hematoxylin and eosin. The images were captured using a BX61 Olympus photomicroscope equipped with a digital camera and a 20× objective lens.

### Quantitation of sterols

For sterol analysis, approximately equal amount of the liver or brain tissues was weighed and homogenized in 1 ml of PBS, pH 7.4. The resultant homogenates were centrifuged twice at 1,000 *g* each for 15 min to remove the tissue debris, followed by protein estimation using a BCA protein assay kit (23225; Pierce Biotechnology, Rockford, IL), so as to achieve a final protein amount of 500 μg for each sample. Aliquots (100–200 μL each) of the resultant tissue lysates and 10 μL of plasma or bile were used for extraction and analysis of sterols. The sterols were derivatized with *N, N*-dimethylglycine so that they could be detected using ESI mass spectrometry. The method was based on published procedures for similar compounds (27, 28), the details of which will be published separately (Tallman, K. A. and Porter, N. A., manuscript in preparation). In brief, a stock solution of deuterated standards was made containing 30 μM of *d*_*7*_-Chol, *d*_*6*_-Lan, *d*_*7*_-dHLan, and 3μM *d*_*7*_-7-DHC, *d*_*7*_-8-DHC, *d*_*6*_-Des, *d*_*6*_-DHD, *d*_*7*_-Lath, *d*_*6*_-DHL, *d*_*7*_-Zyme, *d*_*6*_-Zym, *d*_*7*_-14d-Zyme, *d*_*6*_-14d-Zym in methanol (MeOH). The standard stock solution contained 1% Et_3_N, BHT, and PPh_3_ to prevent isomerization and/or oxidation. The concentration of the standard was in the linear range of absolute endogenous sterol values detected by mass spectrometry. Vitamin D3 and 25-OH vitamin D3 were quantified relative to *d*_*7*_-Chol using response factors of 1.0 and 11.2, respectively. To each sample was added the standard mixture (10 μL), CHCl_3_:MeOH (600 μL, 2:1), and saline (300 μL). The sample was vortexed well and centrifuged at 5,000 rpm (2,152 *g*) for 1 min to separate the layers. The CHCl_3_ layer was transferred to a new vial and dried under vacuum on a SpeedVac.

The subsequent steps include derivatization of the extracted sterols and LC-MS/MS analysis of sterols. Derivatizing reagent was freshly prepared with 2-methyl-6-nitrobenzoic anhydride (20 mg), *N, N*-dimethylglycine (14 mg), DMAP (6 mg), and Et_3_N (0.1 mL) in anhydrous CHCl_3_ (0.9 mL). To each sample was added derivatizing reagent (100 μL) and allowed to react at room temperature for 30 min. The samples were dried under vacuum and subsequently dissolved in MeOH (100 μL) for LC-MS/MS analysis. Samples were analyzed on an Acquity UPLC system equipped with ANSI-compliant well plate holder. The sterols (10 μL injection) were separated on an Agilent Poroshell® EC-C18 (10 cm × 2.1 mm, 1.9 μm) with CH_3_CN:MeOH:H_2_O, 70:25:5 (0.01% (v) formic acid, 1 mM NH_4_OAc) mobile phase at a column temperature of 40°C. The flow rate was 400 μL/min for 11.5 min, then ramped to 600 μL/min at 11.6 min with a total run time of 16 min. A TSQ Quantum Ultra tandem mass spectrometer (ThermoFisher) was used for MS detections, and data were acquired with a Finnigan XCalibur® software package. Selected reaction monitoring (SRM) of the DMG-sterol derivatives was acquired in the positive ion mode using electrospray ionization (ESI). MS parameters were optimized using DMG-Chol and were as follows: spray voltage at 4500 V, capillary temperature at 300°C, auxiliary nitrogen gas pressure at 55 psi, and sheath gas pressure at 60 psi. Collision energy (CE) was optimized for each sterol under a collision gas pressure of 1.5 mTorr. Endogenous sterol levels were quantified based on the known internal standard amount and then normalized to mg protein for tissues (liver, brain, heart, kidney) and volume for biological fluids (plasma, bile). A representative chromatogram with SRMs for the sterol analysis is presented in supplemental Figure S1.

### Tolerance tests and hepatic triglyceride secretion assay

Glucose (GTT) and insulin (ITT) tolerance tests were performed by measurement of blood glucose at 0, 30, 60, 90 and 120 min using OneTouch® Ultra® glucometer (LifeScan Inc., Milpitas, CA) after intraperitoneal administration of glucose at 2 g/kg dose or human insulin, Humulin-R (HI-213; Lilly, Indianapolis, IN) at 0.6 IU/kg dose to mice after 4 h of fasting. Hepatic triglyceride secretion rates were determined by measurement of plasma triglycerides using a Triglyceride Assay Kit (TR22421; Thermo Fischer Scientific, Middletown, VA) with blood collected at 0, 30, 60, 90, and 120 min after *i*.*p*. injection of Poloxamer 407 (P-407, a.k.a. Pluronic® F127) at 1 g/kg dose (P2443; Sigma-Aldrich, St. Louis, MO) (29, 30).

### RNA-seq analyses

RNA was isolated from liver tissues (n = 3 per group) by column purification using a Qiagen RNeasy® Kit (74104; Qiagen Inc, Germantown, MD) and submitted for next-generation sequencing by the DNA Sequencing and Genotyping Core Facility of Cincinnati Children's Hospital Medical Center (CCHMC). In brief, 150–300 ng of total RNA determined by Invitrogen Qubit high-sensitivity spectrofluorometric measurement was poly-A selected and reverse transcribed using Illumina's TruSeq stranded mRNA library preparation kit. Each sample can be fitted with one of 96 adapters containing a different 8 base molecular barcode for high-level multiplexing. After 15 cycles of PCR amplification, completed libraries were sequenced on an Illumina NovaSeq 6000, generating 20 million or more high-quality 100-base long paired end reads per sample.

The first step in analysis of the FASTQ data files includes the quality control steps to determine overall quality of the reads. Upon passing basic quality matrices, the reads were trimmed to remove adapters and low-quality reads using Trimmomatic. The trimmed reads were then mapped to the mouse (mm10) reference genome. Hisat2 was used for reference alignment. In the next step, transcript/gene abundance was determined using “Kallisto.” We first created a transcriptome index in Kallisto using Ensemble cDNA sequences for mouse (mm10). This index was then used to quantify transcript abundance in raw counts and transcript per million (TPM). The quantified count matrix was then used for determining differential gene expression between control and KO samples. An R package called RUVSeq was used to perform the differential gene expression analysis between the groups of samples, raw counts obtained from Kallisto are used as input. The significant differentially expressed genes can obtained by using a fold change cutoff of 2 and adjusted *P*-value/*P*-value cutoff of ≤ 0.05 (LogFC = 1, FDR = 0.05). Downstream functional annotation of gene of interest and/or significantly dysregulated in the experiment is determined using gene ontology (cellular components, molecular function, and biological process) and pathway analysis. A detailed functional annotation and pathway analysis was performed using TOPPFUN and TOPPGENE (31).

### QPCR analyses

Total RNA was isolated from liver tissues (n = 5 per group) by column purification using a Qiagen RNeasy® Kit (74104; Qiagen Inc, Germantown, MD) and was reverse transcribed to cDNA using High-Capacity RNA-to-cDNA™ Kit (4387406; ABI, Foster City, CA). Quantitative assessment of target gene expression was performed on Applied Biosystems 7300 Real-Time PCR system (Applied Biosystems, Foster City, CA) using commercially available Taqman real-time PCR probes (ABI Biosystems). All reactions were performed in triplicate, and *Atp5po* was used as a housekeeping gene for data normalization to compensate for variations between input RNA amounts and data was analyzed using comparative C_T_ method.

### Statistical analyses

All data are shown as mean ± SD. Sex- and age-matched groups were compared using either Student's *t*-test or multiple *t*-test or Mann-Whitney U test (Mann-Whitney-Wilcoxon rank-sum test) only when the comparison benefited from a statistical test of significance. A *P*-value < 0.05 was considered statistically significant. Prior to analyses, the data were assessed as to whether the values obtained came from a Gaussian distribution using Shapiro-Wilk's/Kolmogorov-Smirnov/D'Agostino and Pearson omnibus normality tests, respectively.

## Results

### Generation of liver-specific *Dhcr7* KO mice

**Fig. 1:**
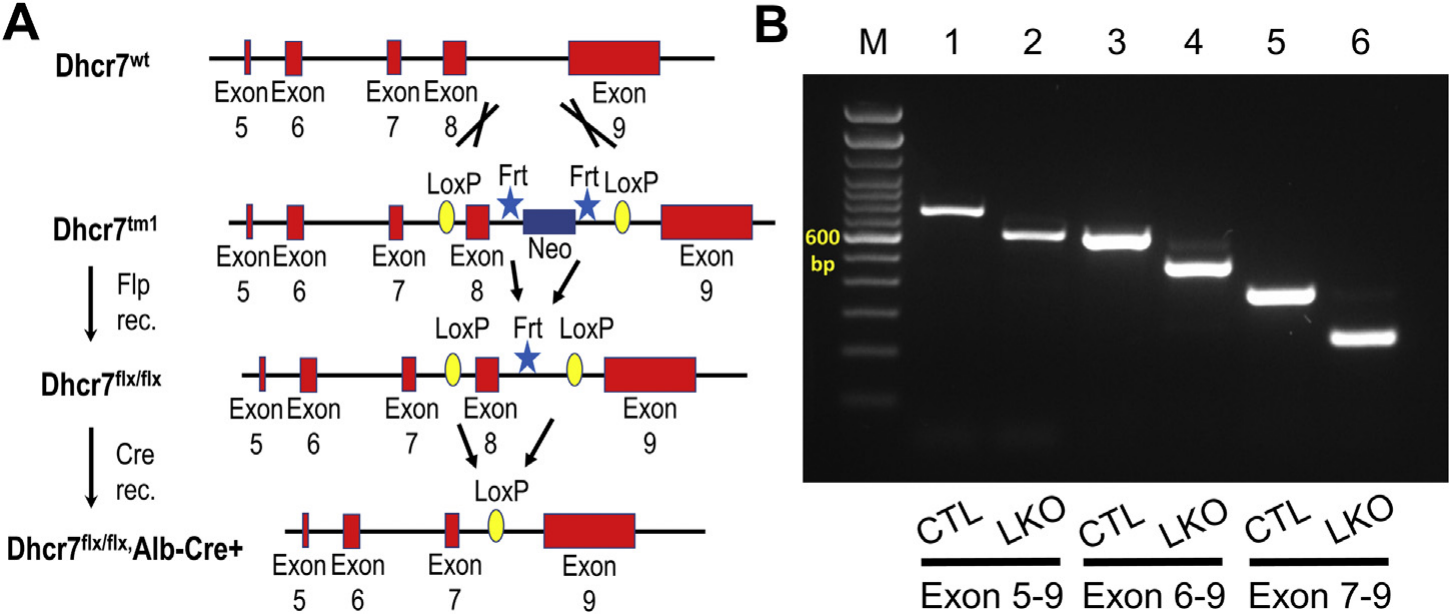
Verification of *Dhcr7* gene deletion in *Dhcr7*^flx/flx,^Alb-Cre+ mice liver. A: A schematic representation of the targeting vector used for generation of *Dhcr7* conditional KO. Reverse transcription PCR analysis of liver RNA (B) to detect bona fide exon splicing showed a consistently smaller PCR product in the liver-specific knockout (LKO) mice spanning exon 8 compared with control (CTL) mice, and this size difference matches the size of exon 8.

The construct that was employed to target *Dhcr7* for conditional knockout is detailed in Fig. 1A. We performed RT-PCR analysis of exon splicing and genomic DNA isolated from the liver to verify the deletion of *Dhcr7* exon 8 in the mature mRNA, as there is not a validated antibody available to confirm the absence of the protein (Fig. 1B). Total RNA isolated from the livers of *Dhcr7*^flx/flx^ and *Dhcr7*^*L-KO*^ mice were used to generate cDNA. The regions from exons 5–9, 6–9, or 7–9 were amplified and confirmed smaller-sized products in *Dhcr7*^*L-KO*^ (tracks 2, 4, and 6) liver when compared with *Dhcr7*^flx/flx^,Cre− (tracks 1, 3 and 5) livers, indicating deletion of the 132 bp exon 8 (Fig. 1B). Theoretically, deletion of exon 8 can result in an in-frame translation of a protein when exon 7 is joined to exon 9, although the critical enzymatic domain would be disrupted. To verify that loss of exon 8 can lead to a null phenotype, we generated germ-line exon-deleted mice (Dhcr7^Δ8/+^) and inter-crossed these; of 14 pups born from 2 litters, 6 were Dhcr7^Δ8/Δ8^, 2 were Dhcr7^+/+^, and six were Dhcr7^Δ8/+^. All Dhcr7^Δ8/Δ8^ died within 24 h of birth, whereas none of the other genotypes died spontaneously, thus confirming that the loss of exon 8 does not result in a hypomorph and behaves as a null allele.

### Deletion of *Dhcr7* in liver does not disrupt development or metabolism

**Fig. 2:**
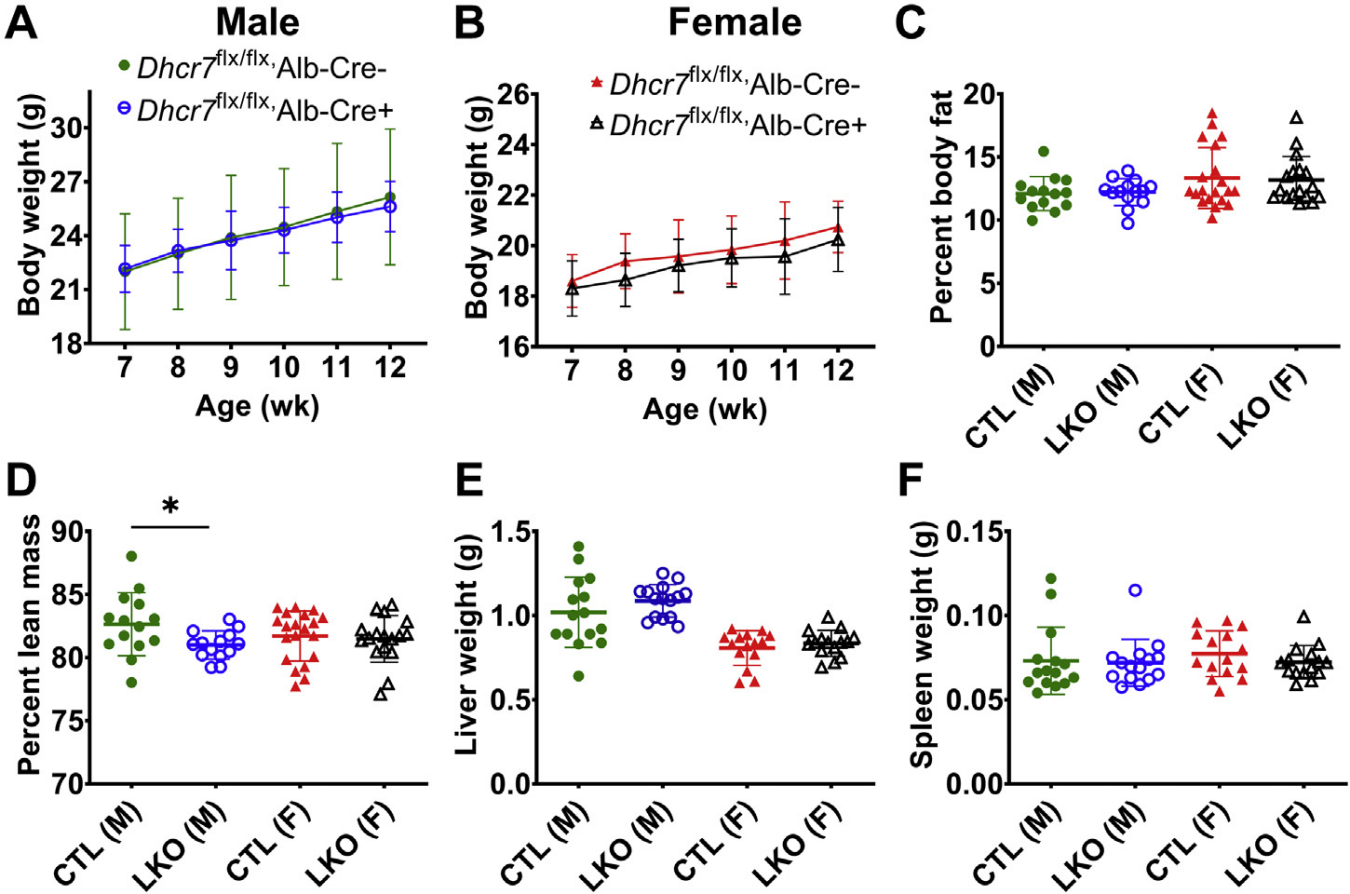
Growth curves, body composition, and organ weights in liver-specific knockout (LKO) mice and sex- and age-matched controls (CTL). The growth velocity of male (A) and female mice (B) was comparable between the liver-specific knockout mice (*Dhcr7*^flx/flx^,Cre+) compared with control mice (*Dhcr7*^flx/flx^,Cre−), and although no differences in body fat (C) were noted, male knockout mice showed a statistically significant lower lean body mass (D), though these differences are quite small (measured at 10–14 weeks of age). There were no differences in the liver (E) or spleen (F) weights at sacrifice at 13–14 weeks of age. Comparisons were made only within each sex. Males are represented by circles, and females by triangles. LKO mice are represented by open symbols and CTL mice by closed symbols. Bars denote the mean ± 1 SD. For body weight measurements in weeks 7–10, n = 27 male, 26 female *Dhcr7*^flx/flx^,Cre+ mice and n = 9 males, 11 female *Dhcr7*^flx/flx^,Cre− mice; in weeks 11–12, n = 18 male, 16–21 female *Dhcr7*^flx/flx^,Cre+ mice and n = 6 males, 5–6 female *Dhcr7*^flx/flx^,Cre− mice. For body composition measurements, n = 14 male LKO and CTL mice, 17 female LKO and 21 female CTL mice. For the liver and spleen weights, n = 15 mice per group.

*Dhcr7*^*L-KO*^ (Dhcr7^flx/flx^,Alb-Cre^+^) mice did not exhibit any noticeable dysmorphology or change in fertility or survival. Body weights (Fig. 2A, B), percent body fat (Fig. 2C), and lean mass (Fig. 2D) were similar among all genotypes. In addition, the weights of the liver, spleen (Fig. 2E, F), heart (supplemental Fig. S2F), and kidney (supplemental Fig. S3F) were also comparable among all genotypes at sacrifice.

Given the role of the liver in maintaining systemic glucose and lipid homeostasis (32, 33), we measured glucose and insulin tolerance and found these to be unaltered (supplemental Fig. S4A, B). Similarly, triglyceride secretion rates were not significantly different between *Dhcr7*^*L-KO*^ and control mice (supplemental Fig. S4C).

### *Dhcr7*^*L-KO*^ mice showed elevated levels of circulatory 7-dehydrocholesterol

We first profiled plasma and biliary sterols using small subsets of heterozygous mice, with and without Cre recombinase (*Dhcr7*^flx/wt,^Alb-Cre^+^ and *Dhcr7*^flx/wt,^Alb-Cre^−^), to see if loss of one *Dhcr7* allele in the liver led to biochemical differences. Almost all sterols assessed in both male and female mice remained at baseline and comparable between groups (supplemental Fig. S5), confirming that a single copy of *Dhcr7* was sufficient to maintain a normal biochemical phenotype. Therefore, in subsequent biochemical experiments, both genotypes were included in the control cohort along with *Dhcr7*^flx/flx,^Alb-Cre− mice. However, for RNA-Seq experiments (see later), the comparison was kept strictly between *Dhcr7*^*L-KO*^ and littermates, which were *Dhcr7*^flx/flx,^Alb-Cre−.

**Fig. 3:**
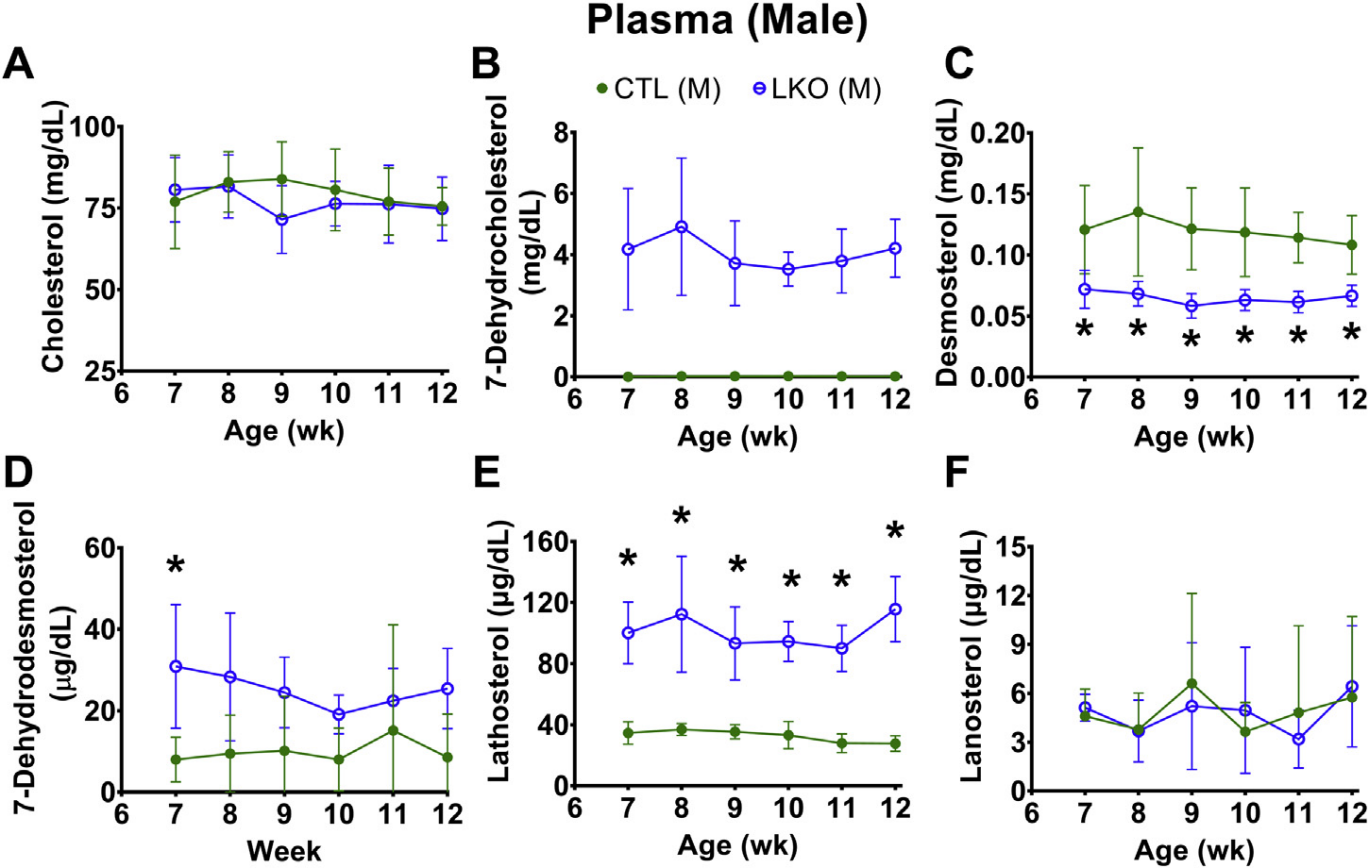
Plasma sterol profiles in male liver-specific knockout (LKO) mice compared with controls (CTL). Weekly profiles of cholesterol (A), 7-dehydrocholesterol (B), desmosterol (C), 7-dehydrodesmosterol (D), lathosterol (E), and lanosterol (F) are shown. Liver knockout mice are represented by open circles and control mice by closed circles. As expected, 7-DHC (B) was characteristically elevated and desmosterol levels depressed (panel C) in liver-specific knockouts. In controls, 7-DHC levels were undetectable at all time points (B). Lathosterol levels were also statistically elevated at all time points in liver-specific male mice. Error bars denote ± 1 SD. (∗*P* < 0.05 vs. controls). N = 5–6 per group.

**Fig. 4:**
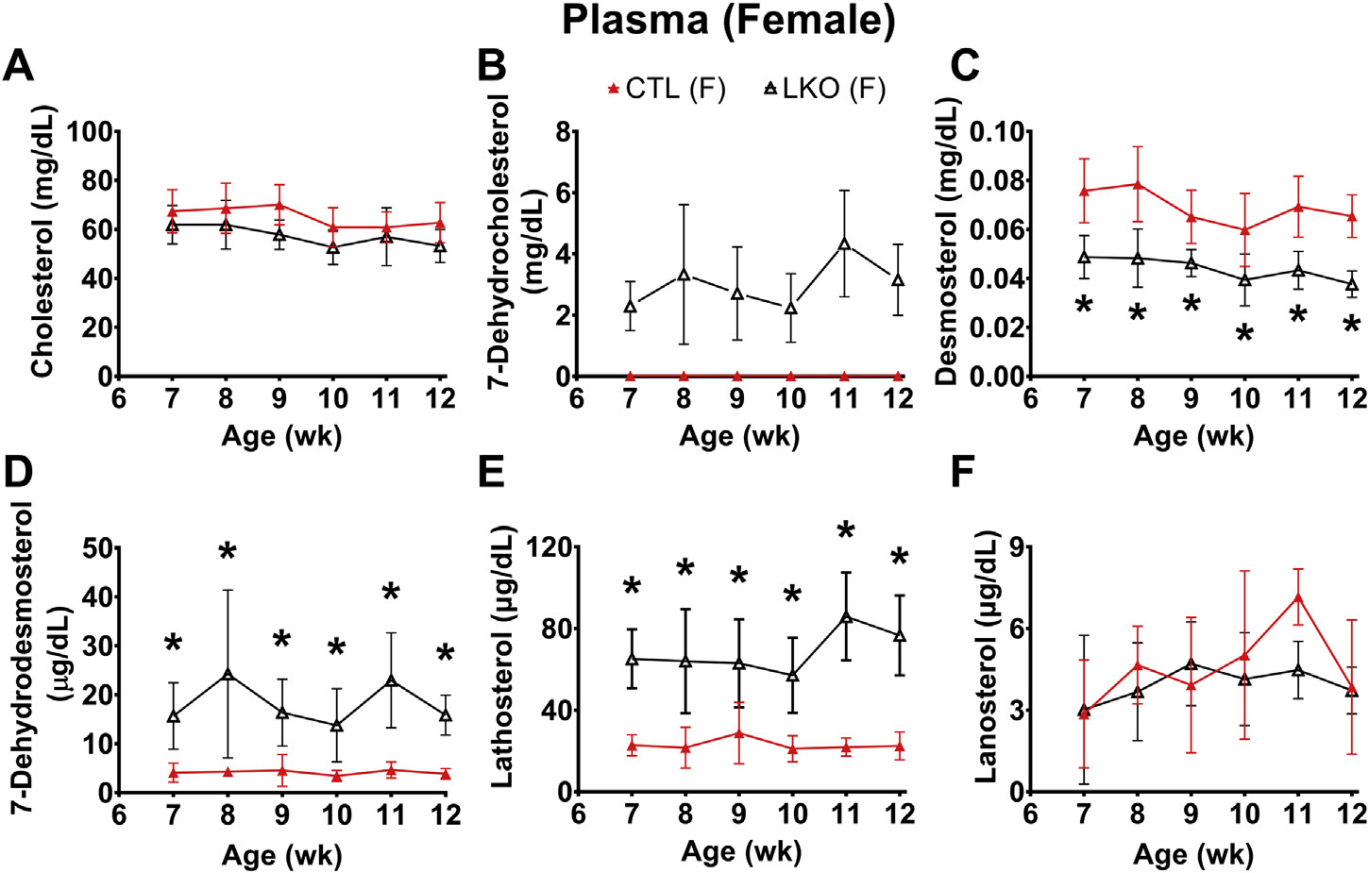
Plasma sterol profiles in female liver-specific knockout (LKO) mice compared with controls (CTL). Weekly profiles of cholesterol (A), 7-dehydrocholesterol (B), desmosterol (C), 7-dehydrodesmosterol (D), lathosterol (E), and lanosterol (F) are shown. Liver knockout mice are represented by open triangles and control mice by closed triangles. As expected, 7-DHC (B) was characteristically elevated and desmosterol levels depressed (C) in liver-specific knockouts. In controls, 7-DHC levels were undetectable at all time points (B). Lathosterol levels were also statistically elevated at all time points in liver-specific female mice. Error bars denote ± 1 SD. (∗*P* < 0.05 vs. controls). N = 6 per group.

Assessment of weekly plasma sterol profiles showed that liver-specific deletion of *Dhcr7* did not alter plasma cholesterol levels (Figs. 3A and 4A), but did result in an elevated steady-state plasma level of the precursor 7-DHC (Figs. 3B and 4B) and its isomer 8-DHC (supplemental Fig. S6A, B). One step earlier in the Kandutsch-Russell pathway, levels of lathosterol (cholest-7-en-3β–ol; Figs. 3E and 4E) were markedly elevated, while those of lanosterol (4α,4β,14α-trimethyl-cholesta-8,14-dien-3β-ol; Figs. 3F and 4F) remained comparable between LKO and control animals. Despite the elevated levels of plasma 7-DHC, changes in levels of vitamin D3 and 25OH-vitamin D3 were not significant (supplemental Fig. S6C, F). In the Bloch pathway, there was a significant decrease in plasma desmosterol in both male (Fig. 3C) and female *Dhcr7*^*L-KO*^ mice (Fig. 4C), and the levels of its immediate precursor, 7-dehydrodesmosterol (DHD), were found to be consistently increased only in females (Figs. 3D and 4D). However, the differences in levels of 24-dehydrolathosterol (DHL) were increased in males but were not consistent over time in female LKO mice (supplemental Fig. S6D, E).

### Elevated levels of 7-DHC in *Dhcr7*^*L-KO*^ liver, but not in the brain

**Fig. 5:**
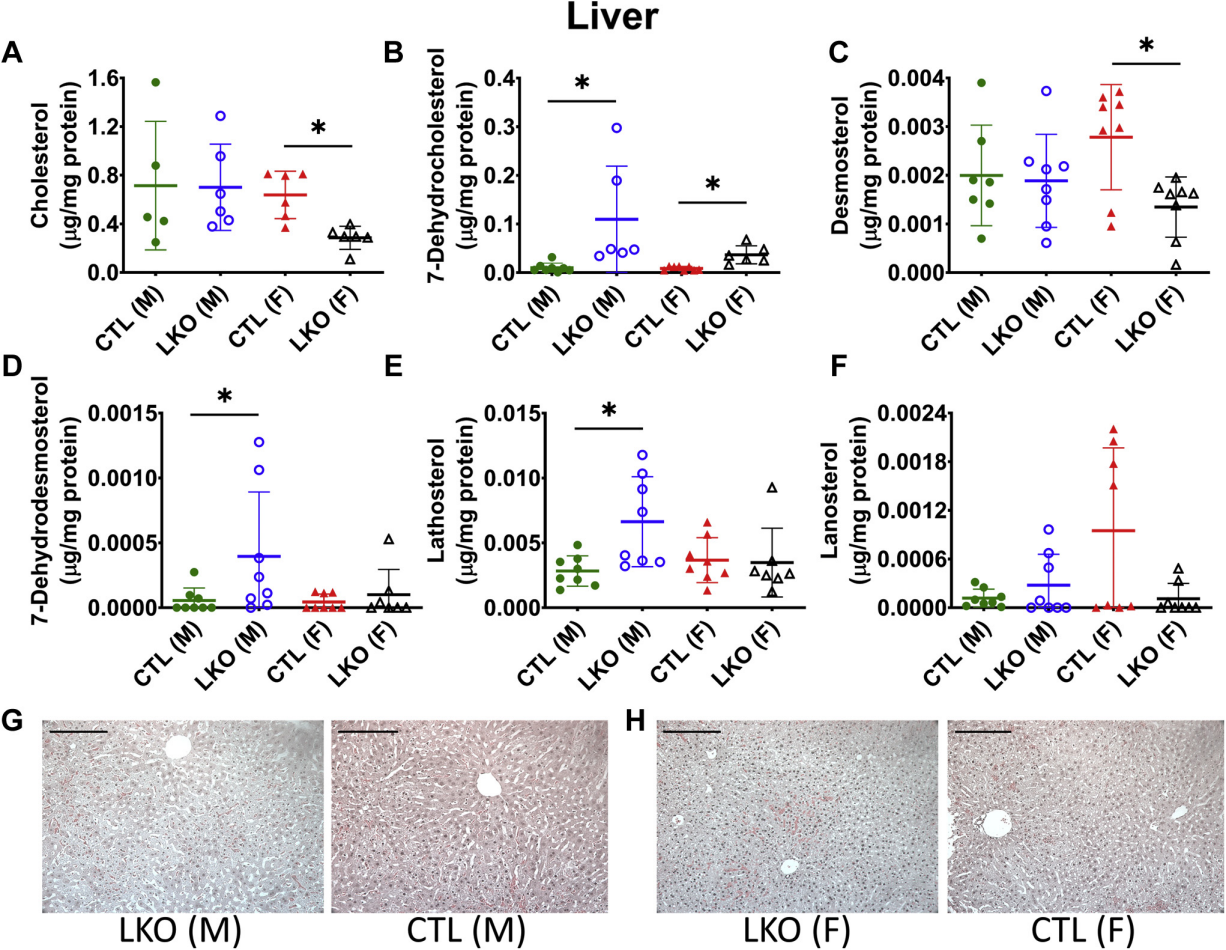
Effect of liver-specific deletion of *Dhcr7* on hepatic sterol content and liver architecture. Hepatic cholesterol (A), 7-DHC (B), desmosterol (C), 7-dehydrodesmosterol (D), lathosterol (E), and lanosterol (F) levels, and H&E stained sections of the liver tissue (G, H) from LKO and CTL mice are shown. Male livers showed no differences in cholesterol levels, but cholesterol levels were lower in livers of female LKO mice (A). As expected, 7-DHC was significantly higher in both male and female knockout livers (B), and while there were some statistical differences in other precursor sterols, there was a lack of a consistent pattern and there is considerable variability between samples (see text for Discussion). Liver micro-architecture was not affected by hepatic loss of *Dhcr7* in either male or female mice (G, H). Males are represented by circles, and females by triangles. LKO mice are represented by open symbols and CTL mice by closed symbols. Bars denote the mean ± 1 SD, (∗*P* < 0.05 vs. sex-matched controls). Scale bar in H&E images represents 100 μm distance. N = 5–8 per group.

**Fig. 6:**
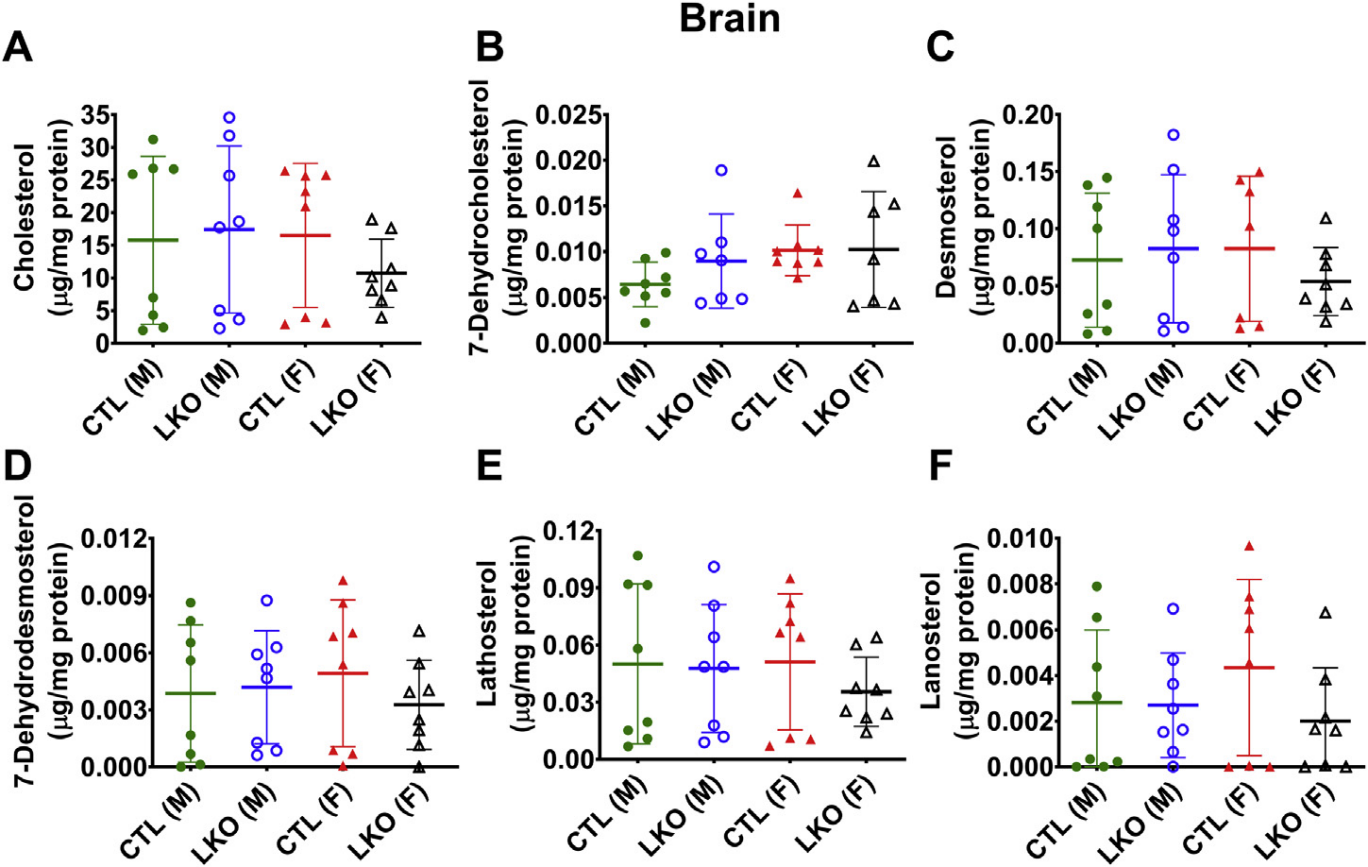
Effect of liver-specific deletion of *Dhcr7* on brain sterol content. Levels of cholesterol (A), 7-DHC (B), 8-DHC (C), desmosterol (D), lathosterol (E), and lanosterol (F), were not altered by liver specific loss of *Dhcr7*. Males are represented by circles, and females by triangles. LKO mice are represented by open symbols and CTL mice by closed symbols. Bars denote the mean ± 1 SD. N = 7–8 per group.

As expected with a liver-specific deletion, altered sterol profiles were observed in the livers of *Dhcr7*^*L-KO*^ mice (Fig. 5), but not in their brains (Fig. 6) due to restriction by the blood-brain barrier. Levels of 7-DHC (Fig. 5B) and 8-DHC (supplemental Fig. S7A) were elevated in livers of both male and female *Dhcr7*^*L-KO*^ mice when compared with controls, whereas levels of cholesterol and desmosterol were decreased only in livers of female *Dhcr7*^*L-KO*^ mice (Fig. 5A, C). Conversely, male *Dhcr7*^*L-KO*^ mice demonstrated elevated levels of lathosterol and DHD in liver, while there was no significant difference in female LKO mice (Fig. 5D, E). The levels of lanosterol (Fig. 5F) remained comparable between liver samples from LKO versus control mice. None of the measured sterols differed significantly between the brains of *Dhcr7*^*L-KO*^ and control mice (Fig. 6A, F and supplemental Fig. S7B, C). Analysis of the heart and kidney of LKO mice showed accumulation of K-R pathway precursors, similar to plasma levels, but did not replicate the changes in the Bloch pathway precursors (supplemental Figs. S2 and S3).

### Hepatobiliary secretion of sterols in *Dhcr7*^*L-KO*^ mice

**Fig. 7:**
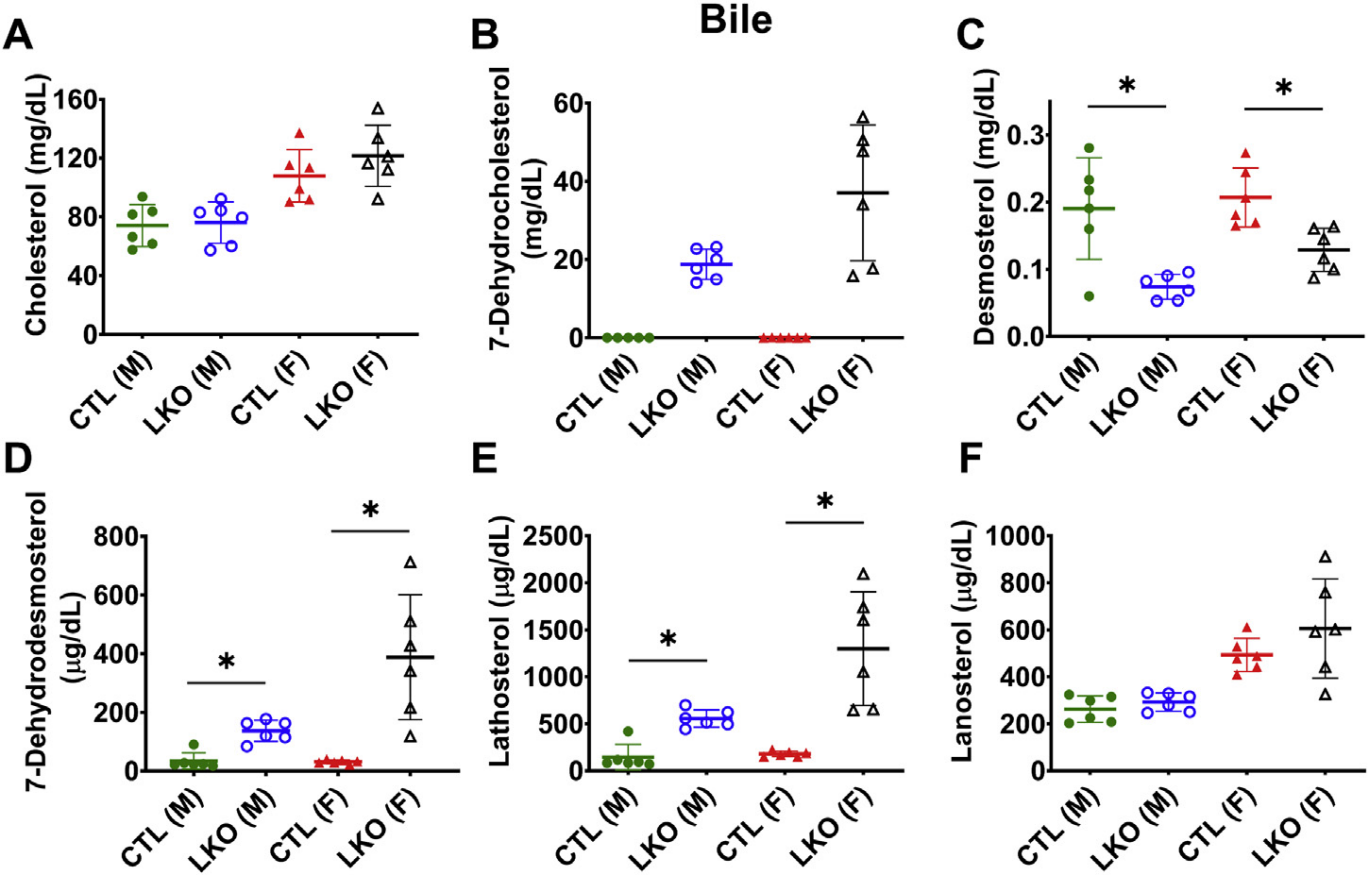
Biliary sterol profiles in liver-specific *Dhcr7* knockout mice. Although biliary cholesterol was unaltered in liver-specific knockout mice compared with sex-matched controls (A), there was significant excretion of 7-DHC into bile of LKO mice and undetectable levels in CTL mice (B). Bile of LKO mice also demonstrated decreased desmosterol (C), increased 7-dehydrodesmosterol (D) and lathosterol (E), but no difference in lanosterol (F). Males are represented by circles, and females by triangles. LKO mice are represented by open symbols and CTL mice by closed symbols. Bars denote the mean ± 1 SD. (∗*P* < 0.05 vs. respective controls). N = 5–6 per group.

**Fig. 8:**
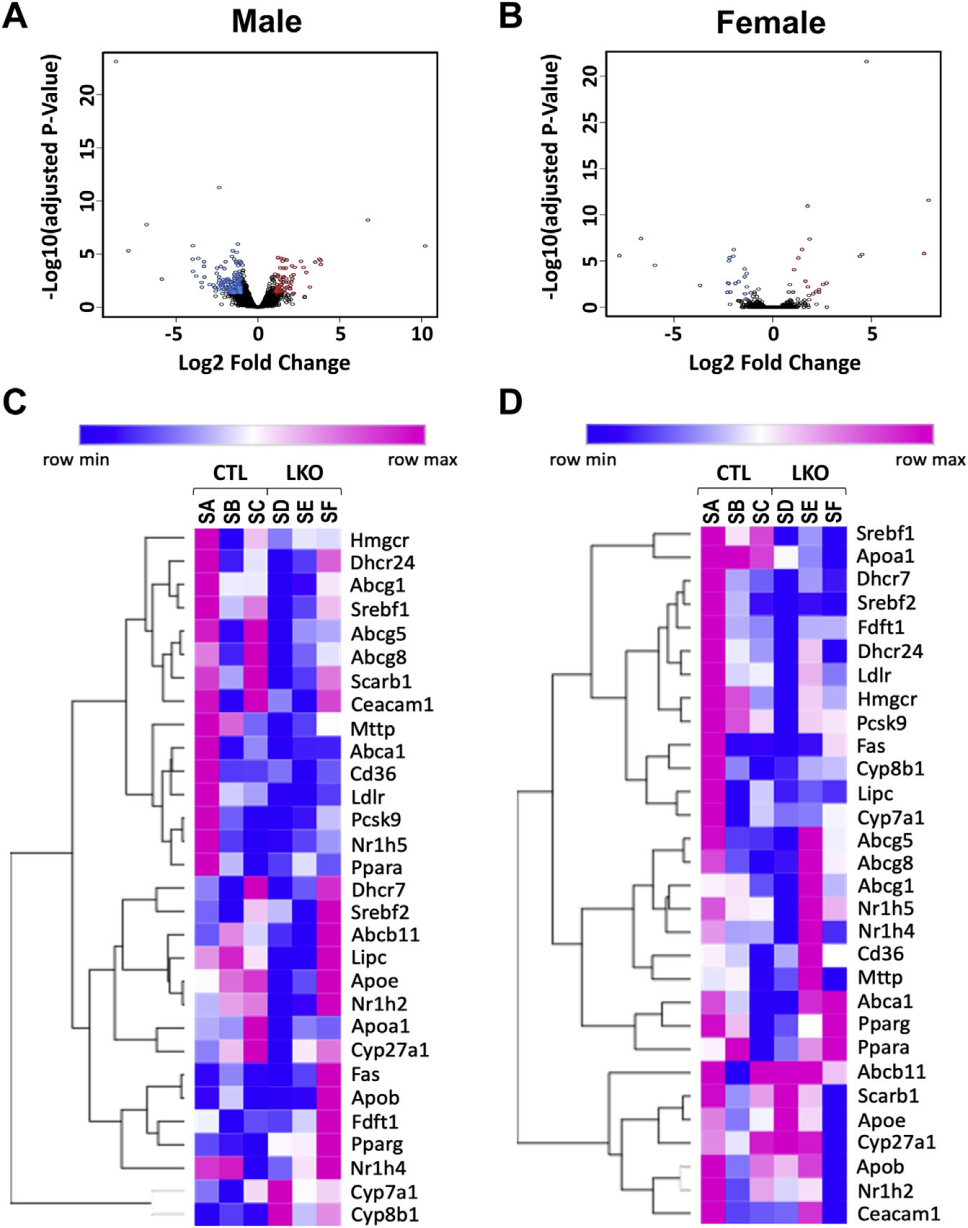
Volcano plots and heatmaps of liver gene expression analyses using RNASeq. Volcano plots (A, B) demonstrate the overall pattern of differential gene expression in male and female LKO liver respectively and heatmaps (C, D) show the relative changes in gene expression (as in TPM's) for 30 key genes participating in lipid homeostasis. Red, blue, or black color for each circle in a volcano plot corresponds to the genes significantly upregulated, downregulated, and unchanged in *Dhcr7*^flx/flx,^Alb-Cre+ (LKO) compared with *Dhcr7*^flx/flx,^Alb-Cre− (control) mice of same sex. In heatmaps, a change toward blue from red denotes the relative gene expression scaled from lowest to highest for each row. Samples SA, SB, and SC are from control livers and samples SD, SE, and SF are from LKO livers (C, D). There were not many large differences in gene expression and no consistent patterns of changes between LKO livers and control mice or either sex. N = 3 per group.

Secretion of excess sterols directly into bile or indirectly as bile acids is the preferred route for sterol excretion from the body (34, 35). The biliary sterol profile of *Dhcr7*^*L-KO*^ mice more closely mirrored the plasma sterol levels than the hepatic sterol levels. The cholesterol content of bile from *Dhcr7*^*L-KO*^ mice was similar to control animals (Fig. 7A), but 7-DHC (Fig. 7B) and 8-DHC levels (supplemental Fig. S7D) in bile were significantly elevated, and desmosterol secretion into bile was decreased in both male and female *Dhcr7*^*L-KO*^ mice when compared with their respective controls (Fig. 7C). Earlier precursors DHD (Fig. 7D), lathosterol (Fig. 7E), and DHL (supplemental Fig. S7E) were also elevated, whereas lanosterol was unchanged (Fig. 7F) in *Dhcr7*^*L-KO*^ mice when compared with their respective controls.

Subsequently, we also analyzed the levels of total bile acid and phospholipid in both bile and plasma and only found a small increase in levels of biliary bile acids in male *Dhcr7*^*L-KO*^ mice (supplemental Fig. S8A) compared with controls. Phospholipid levels, however, did not differ in bile or plasma (supplemental Fig. S8C, D) as a function of genotype.

### Changes in liver gene expression

Given the observed changes in sterol concentrations with deletion of *Dhcr7* in the liver, we further examined the potential changes in the liver transcriptome using RNA-Seq (Fig. 8). Analysis of raw counts showed differences in a total of 14,587 genes in males and 14,864 genes in females between genetically modified versus. control mouse RNA samples. After applying filters of logFC = 1 and FDR = 0.05 to define significant changes, 231 genes in males and 41 genes in females were identified. Principal component analysis (PCA) using hierarchical clustering showed a higher incidence of similarity between the transcriptomes of *Dhcr7*^*L-KO*^ and *Dhcr7*^flx/flx,^Alb-Cre− livers in females, while males demonstrated a greater difference between groups. This was reflected in the downstream functional annotation of genes in *Dhcr7*^*L-KO*^ mice as determined by gene ontology. In the end, we identified 3 biological processes in females and 60 in males, 7 molecular functions in females and 26 in males, 0 cellular components in females and 13 in males, and 8 pathways in females, and 11 in males. A list of the most changed pathways is included in Table 1, and a limited list of genes exhibiting the greatest increase or decrease in expression is available in the supplemental File S1. Most surprisingly, the genes involved in cholesterol synthesis were not changed. The full data set can be found in NCBI Gene Expression Omnibus (GEO) database (GSE146523).

**Table 1:** Pathways enriched in male and female LKO livers

ID	Term	C^a^	*P*	Genes	Bonferroni	Benjamini	FDR
Male mice							
Mmu 05142	Chagas disease	6	5.23E-03	ADCY1, CCL3, CCL2, GNAI1, SERPINE1, PIK3R5	6.27E-01	6.27E-01	6.29E+00
Mmu 04914	Progesterone-mediated oocyte maturation	5	1.48E-02	ADCY1, PLK1, GNAI1, PKMYT1, PIK3R5	9.40E-01	7.55E-01	1.69E+01
Mmu 04015	Rap1 signaling pathway	7	2.91E-02	PRKD1, ADCY1, GNAI1, FPR1, PIK3R5, ANGPT2, ITGAM	9.96E-01	8.43E-01	3.07E+01
Mmu 05146	Amoebiasis	5	3.87E-02	LAMA4, ADCY1, PIK3R5, COL5A2, ITGAM	9.99E-01	8.44E-01	3.87E+01
Mmu 04670	Leukocyte transendothelial migration	5	3.98E-02	VCAM1, GNAI1, CLDN6, PIK3R5, ITGAM	1.00E+00	7.83E-01	3.96E+01
Female mice							
Mmu 05204	Chemical carcinogenesis	5	2.31E-06	GSTA1, UGT2B38, GSTA2, CYP2C55, SULT2A1	7.16E-05	7.16E-05	1.98E-03
Mmu 00980	Metabolism of xenobiotics by cytochrome P450	4	4.46E-05	GSTA1, UGT2B38, GSTA2, SULT2A1	1.38E-03	6.90E-04	3.83E-02
Mmu 00982	Drug metabolism cytochrome P450	3	2.51E-03	GSTA1, UGT2B38, GSTA2	7.50E-02	2.57E-02	2.14E+00
Mmu 00140	Steroid hormone biosynthesis	3	4.32E-03	UGT2B38, CYP2C55, HSD3B5	1.26E-01	3.30E-02	3.66E+00
Mmu 00040	Pentose and glucuronate interconversions	2	3.80E-02	UGT2B38, AKR1B7	6.99E-01	2.13E-01	2.83E+01

a Refers to count.

**Fig. 9:**
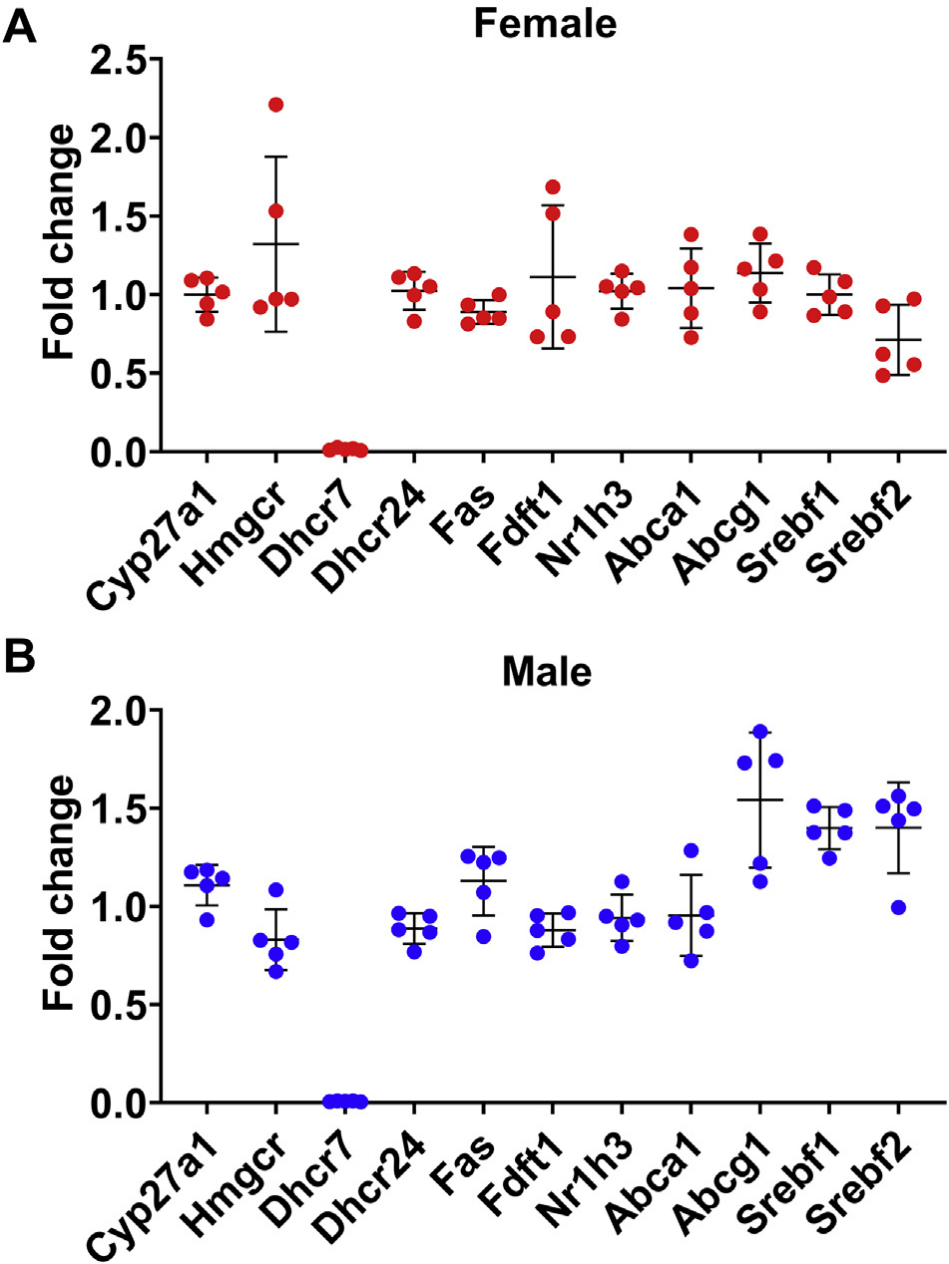
Relative expression of genes involved in cholesterol homeostasis. With the exception of Dhcr7, expression of genes involved in various aspects of cholesterol homeostasis was not significantly different in the livers of LKO mice compared with the livers of control mice (Females: A, Males: B). Each point represents the relative expression of an LKO mouse compared with the mean expression in sex-matched control mice. Bars denote mean ± 1 SD (N = 5 mice per group).

The expression of select genes of interest was confirmed with qPCR, including genes involved in cholesterol homeostasis and targets of liver X receptor (LXR), the latter being known to be activated by desmosterol. After correction for multiple testing, there were no significant differences in gene expression in LKO mice compared with Cre− control mice (Fig. 9).

## Discussion

We have generated the first viable conditional knockout of *Dhcr7* using LoxP-Cre technology and have validated its functionality with a hepatocyte-specific deletion. Initial attempts to generate homozygous floxed mice, utilizing the commercially available EUCOMM construct (Dhcr7^tm1a(EUCOMM)Hmgu^; MGI: 4939763), targeting exon 7, generated successful F0 heterozygous chimeras. However, interbreeding of these heterozygous floxed mice generated only heterozygous and wild-type progeny with a Mendelian ratio of 2:1, respectively, indicating embryonic lethality of homozygotes harboring the floxed *Dhcr7* allele (data not shown). This prompted us to design the current strategy targeting exon 8 using Turbo Knockout ES cells (Fig. 1), which was able to achieve success in germline transmission of the floxed allele. Based upon the crystal structure of a bacterial homolog of mammalian DHCR7, Δ14-sterol reductase (C14SR, EC 1.3.1.70) from *M. alcaliphilum*, we predicted that the targeted exon 8 of murine *Dhcr7* consists of a critical sterol binding pocket (36). Although the loss of exon 8 leads to an in-frame translation of exon 9 with a natural termination, this enzyme should be nonfunctional, even if a protein is expressed. The absence of a validated antibody limits our ability to study this aspect. To verify that loss of exon 8 leads to a null enzyme, we generated heterozygous germ-line exon 8 deleted mice, *Dhcr7*^*Δ8/+*^, and intercrossed these; although *Dhcr7*^*Δ8/+*^ and *Dhcr7*^*+/+*^ pups survived beyond 48 h after birth, all *Dhcr7*^*Δ8/Δ8*^ pups died within 24 h and confirmed that loss of exon 8 results in neonatal lethality, similar to the published global knockout mice.

The liver was chosen as the initial target tissue to validate our new model due to its central role in modulating overall cholesterol homeostasis and its being the major cholesterogenic tissue in vertebrates. Liver-specific *Dhcr7* knockout mice exhibited elevated levels of 7-DHC and 8-DHC and decreased levels of desmosterol in plasma (Figs. 3 and 4), reproducing a biochemical phenotype similar to that observed in SLOS patients (3, 4, 5). Elevated 8-DHC levels, in the setting of elevated 7-DHC levels, are expected because of spontaneous conversion catalyzed by the enzyme 3β-hydroxysterol-Δ^8^,Δ^7^-isomerase (EBP; EC 5.3.3.5) due to increased substrate availability (37). Liver-specific deletion of *Dhcr7* is well tolerated with no obvious phenotypic changes beyond an altered sterol profile and suggests that the changes observed in SLOS are related to loss of *Dhcr7* gene expression in the tissues other than the liver.

The altered sterol profile of plasma was reflected in the sterol profile of bile (Fig. 7), consistent with the observation that hepatobiliary excretion is the preferred mode of clearance of plasma sterols (34, 35). Biliary 7-DHC levels in *Dhcr7*^*L-KO*^ mice were more than 10-fold higher, compared to control mice (Fig. 7). The larger magnitude of increase in 7-DHC likely reflected its position as the immediate precursor for the reaction catalyzed by DHCR7 in the Kandutsch-Russell pathway of cholesterol synthesis. However, the increase in DHD level, which is the immediate precursor for the reaction catalyzed by DHCR7 in the Bloch pathway, was not as large (Figs. 3D and 4D). This suggests a preference for the Kandutsch-Russell pathway of cholesterol synthesis in the liver in mice, or utilization of a potential mixed pathway in which accumulated DHL and DHD are converted to lathosterol and 7-DHC by DHCR24, thereby switching from the Bloch to the K-R pathway (1, 37).

Interestingly, the changes in sterol concentrations in the livers of *Dhcr7*^*L-KO*^ mice appear to be more subtle (Fig. 5) and may be related to the liver's simultaneous roles in cholesterol synthesis and efflux and the complex regulation of these functions. Furthermore, we observed some sex-dependent differences in cholesterol precursor accumulation in the liver and bile. Notably, both *Dhcr7*^*L-KO*^ and control female mice had higher levels of cholesterol and almost all of the measured precursor sterols when compared with their male counterparts (Fig. 7). This is consistent with previous work showing increased bile acid pools and changes in sterol metabolism in female mice (38).

The blood-brain barrier prevents crossover of plasma sterols into the brain, unlike the case for other tissues, such as the heart and kidney. The lack of difference in the sterol profiles of LKO and control mice (Fig. 6) confirms the specificity of the deletion of *Dhcr7* and also indicates that the altered plasma sterol profile has no systemic effect that resulted in a change in brain sterol homeostasis. Conversely, the accumulation of cholesterol precursors in the heart and kidney tissues (supplemental Figs. S2 and S3) demonstrated that the precursors are transported into other tissues, where they can be metabolized into cholesterol and presumably exported. Thus, nonliver synthesis of cholesterol may be able to compensate for the loss of cholesterol synthesis in the liver.

In eukaryotes, cholesterol is the evolutionarily preferred sterol and has many functions including precursor of steroid hormones, incorporation into cell membranes for structural purposes, and modulating various cellular signal transduction processes (39, 40). Pathway analyses of differentially expressed genes from RNA-Seq comparing the gene expression of the livers from male and female *Dhcr7*^*L-KO*^ and *Dhcr7*^flx/flx,^Alb-Cre− identified enrichment in various pathways, which are sex-specific and some common pathways (Table 1). Alteration of the “steroid hormone biosynthesis” pathway is unsurprising, given cholesterol's role as a steroid precursor. Many of the other identified pathways are involved in cell signaling or metabolism and may be related to the role of cholesterol in plasma membranes (41), but would require further investigations to elucidate the mechanism. Most notably missing from the list are the genes related to cholesterol biosynthesis. It is possible that since the plasma cholesterol levels were not significantly reduced, then there may not have been a significant feedback, although the bile secretion pathway was altered in females.

Overall, this work establishes a new conditional knockout model of *Dhcr7*, which will enable detailed studies of cholesterol biosynthesis and the effects of accumulation of cholesterol precursors in specific tissues and cells.

## Conflict of interest

The authors declare that they have no conflicts of interest with the contents of this article.
