# Supplementary material for: Generation and validation of a conditional knockout mouse model for the study of the Smith-Lemli-Opitz syndrome
Source: J Lipid Res. 2020 Nov 22;62:100002. doi: 10.1194/jlr.RA120001101 (PMC7890206; doi:10.1194/jlr.RA120001101)
Supplement: Supplemental Figures and Table [file mmc2.pdf]

# **Generation and validation of a conditional knockout mouse model for the study of the Smith-Lemli-Opitz Syndrome**

Supplementary data

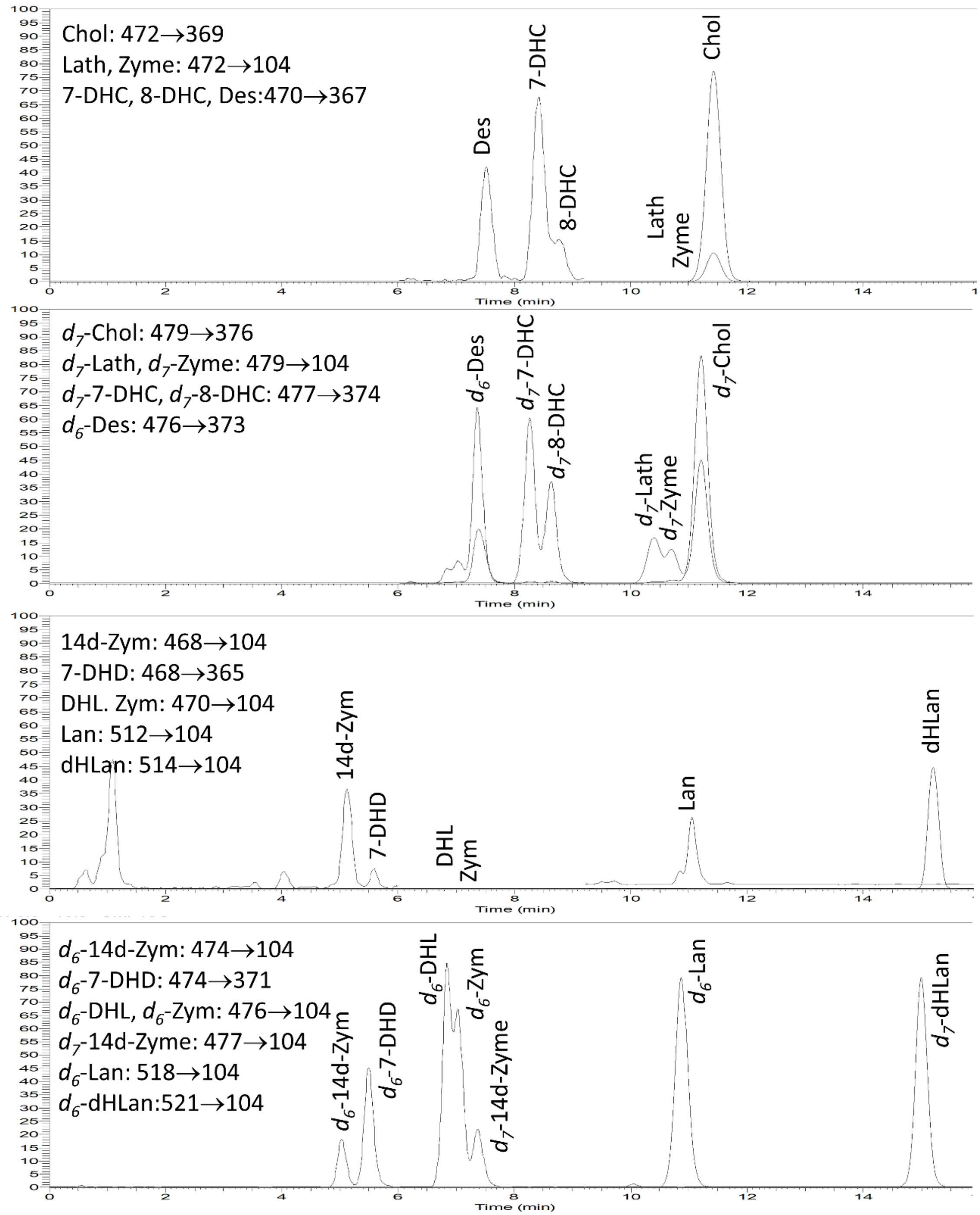


**Figure S1. Representative LC-MS/MS chromatogram for the sterol analysis of liver tissue.** Sterols were separated on an Agilent Poroshell EC-C18 (10 cm x 2.1 mm, 1.9 µm) column, and mass MS detections performed on a TSQ Quantum Ultra tandem mass spectrometer

(ThermoFisher). Data were acquired with a Finnigan Xcalibur software package, and selected reaction monitoring (SRM) of the DMG derivatives was acquired in the positive ion mode using electrospray ionization (ESI). The extracted SRMs shown have been scaled so that all sterols are visible in the chromatogram.

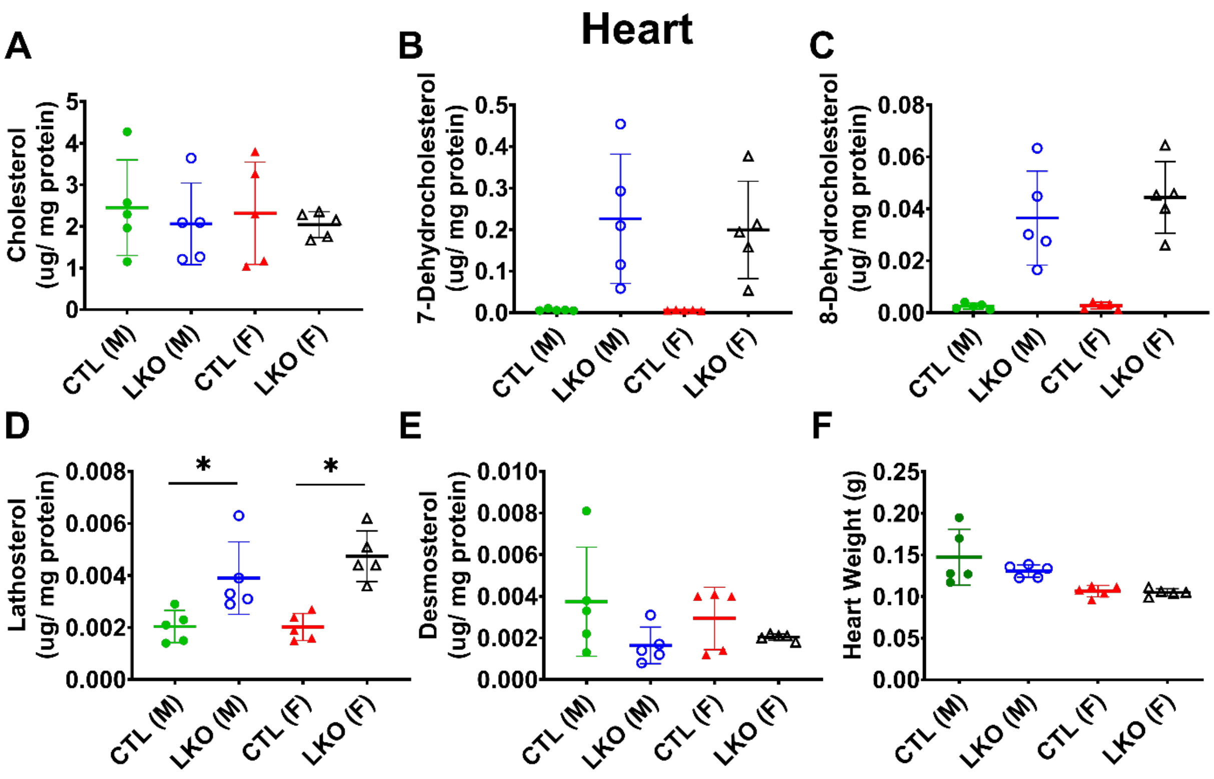


**Figure S2. Effect of liver-specific deletion of *Dhcr7* on sterol content in heart tissue.** Levels of cholesterol (panel A), 7-DHC (panel B), 8-DHC (panel C), lathosterol (panel D), and desmosterol (panel E) in the heart, and heart weight (panel F) of LKO and control mice are shown. LKO mice demonstrated elevated levels of 7-DHC, 8-DHC, and lathosterol, but showed no difference in levels of cholesterol and desmosterol. There was no difference in the size of the hearts of LKO mice compared to control mice. Males are represented by circles, and females by triangles. LKO mice are represented by open symbols and CTL mice by closed symbols. Bars denote the mean ± 1SD, (*=$p<0.05$ vs sex-matched controls). N=5 per group

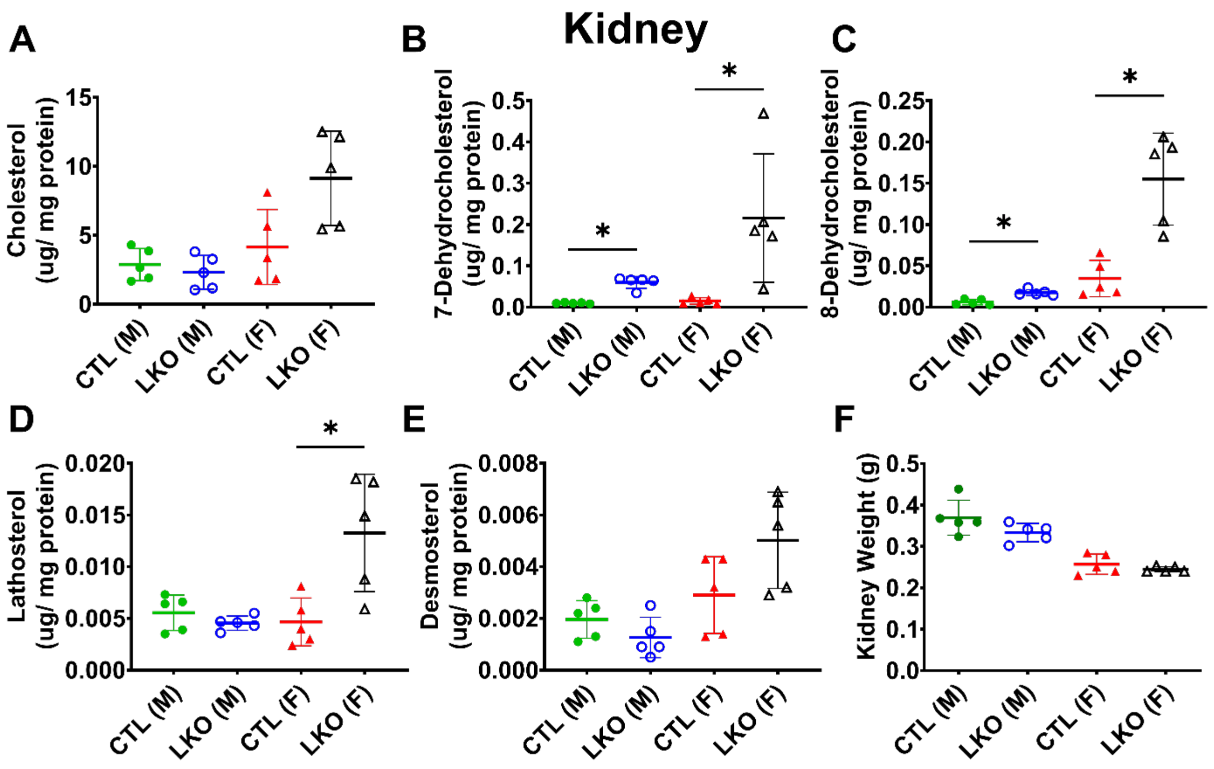


**Figure S3. Effect of liver-specific deletion of *Dhcr7* on sterol content in renal tissue.** Levels of cholesterol (panel A), 7-DHC (panel B), 8-DHC (panel C), lathosterol (panel D), and desmosterol (panel E) in kidney, and kidney weight (panel F) of LKO and control mice are shown. Female LKO mice demonstrated elevated levels of 7-DHC, 8-DHC, and lathosterol, but did not show a significant difference in levels of cholesterol and desmosterol when compared to CTL mice. Levels of 7-DHC and 8-DHC were also elevated in male LKO mice, but in addition to cholesterol and desmosterol, lathosterol also was unchanged. There was no difference in the size of the kidneys of LKO mice compared to control mice. Males are represented by circles, and females by triangles. LKO mice are represented by open symbols and CTL mice by closed symbols. Bars denote the mean ± 1SD, (*=$p<0.05$ vs sex-matched controls). N=5 per group

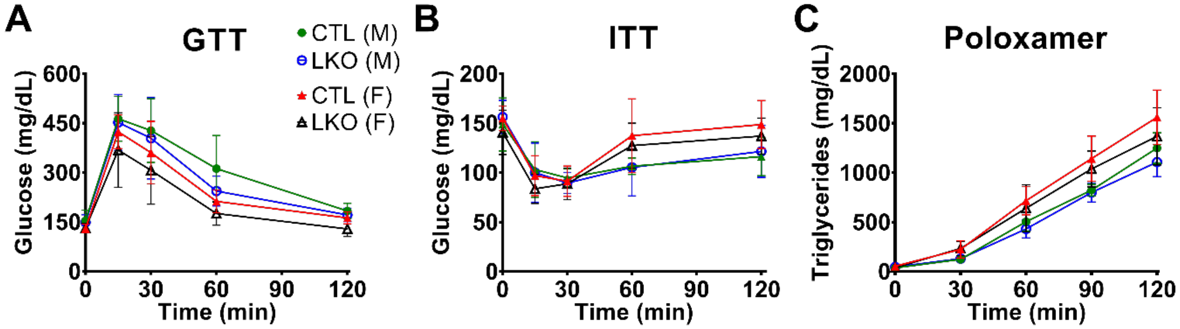


**Figure S4. Glucose homeostasis and hepatic triglyceride secretion rates in control and liver-specific knockout mice.** Mice were injected intraperitoneal with 2g/kg glucose, 0.6units/kg insulin, or 1g/kg Poloxamer-407, and blood collected at 0, 30, 60, and 90mins to assess blood glucose or triglyceride secretion. Glucose and insulin tolerance tests showed no differences between LKO and CTL mice (panels A and B). Hepatic triglyceride secretion did not appear to be affected by loss of *Dhcr7* in hepatocytes (panel C). Males are represented by circles, and females by triangles. LKO mice are represented by open symbols and CTL mice by closed symbols. Bars denote the mean ± 1SD. For GTT, N=9 per group; For ITT, N=6 males per group and 8 females per group, and for triglyceride secretion studies, N=5-6 per group.

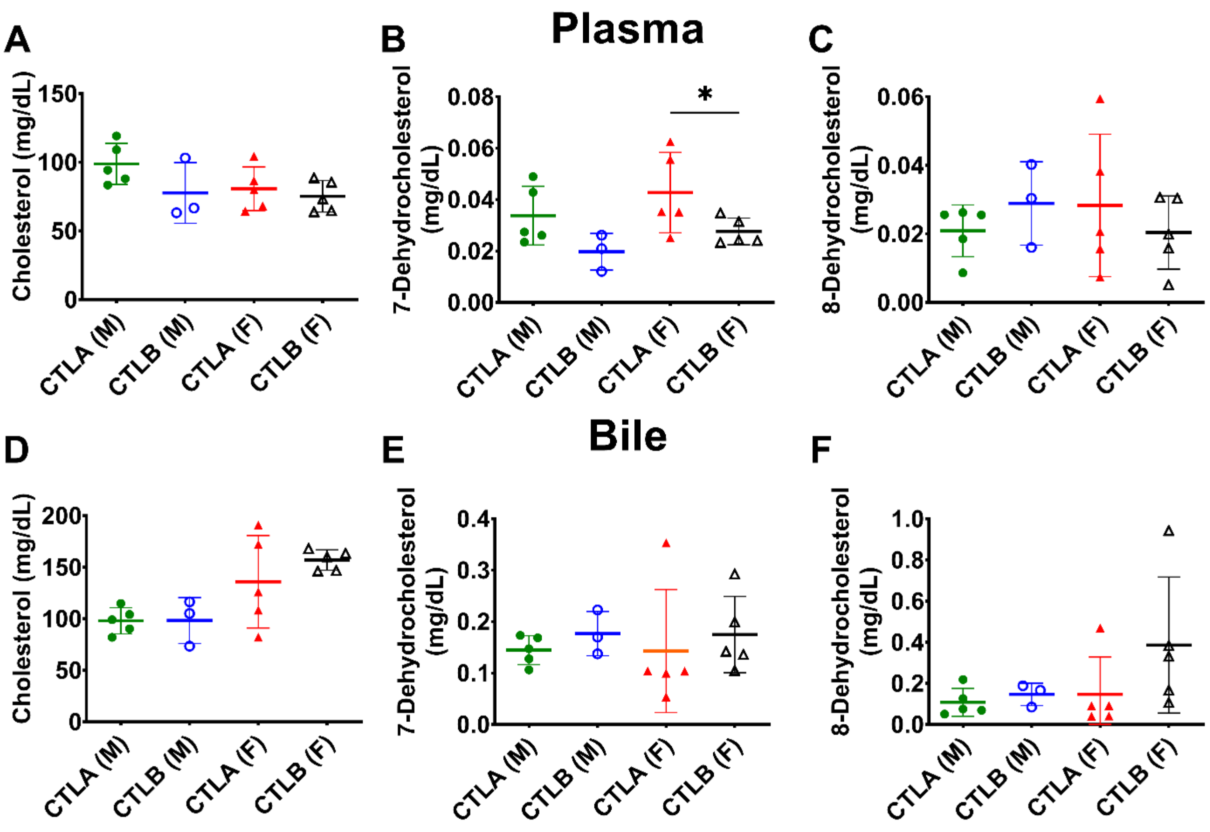


**Figure S5. Sterol profiles of control mice, grouped by genotype.** Sterol profiles were measured in mice with a genotype of *Dhcr7*$^{flx/wt,}$Alb-Cre+ (CTLA) or *Dhcr7*$^{flx/wt,}$Alb-Cre- (CTLB). There were no significant differences in plasma cholesterol and 8-dehydrocholesterol (panel A&C), or biliary cholesterol, 7-dehydrocholesterol (7-DHC) and 8-dehydrocholesterol (panel D-F). Female *Dhcr7*$^{flx/wt,}$Alb-Cre- did demonstrate statistically lower 7-DHC compared to female *Dhcr7*$^{flx/wt,}$Alb-Cre+ but the difference was small and the exhibited plasma 7-DHC levels were very close to the lower limits of detection and therefore may not be biologically meaningful. N=5 mice per group except for male CTLB mice where n=3. Males are represented by circles, and females by triangles. LKO mice are represented by open symbols and CTL mice by closed symbols. Bars denote the mean ± 1SD, (*=p<0.05 vs sex-matched controls).

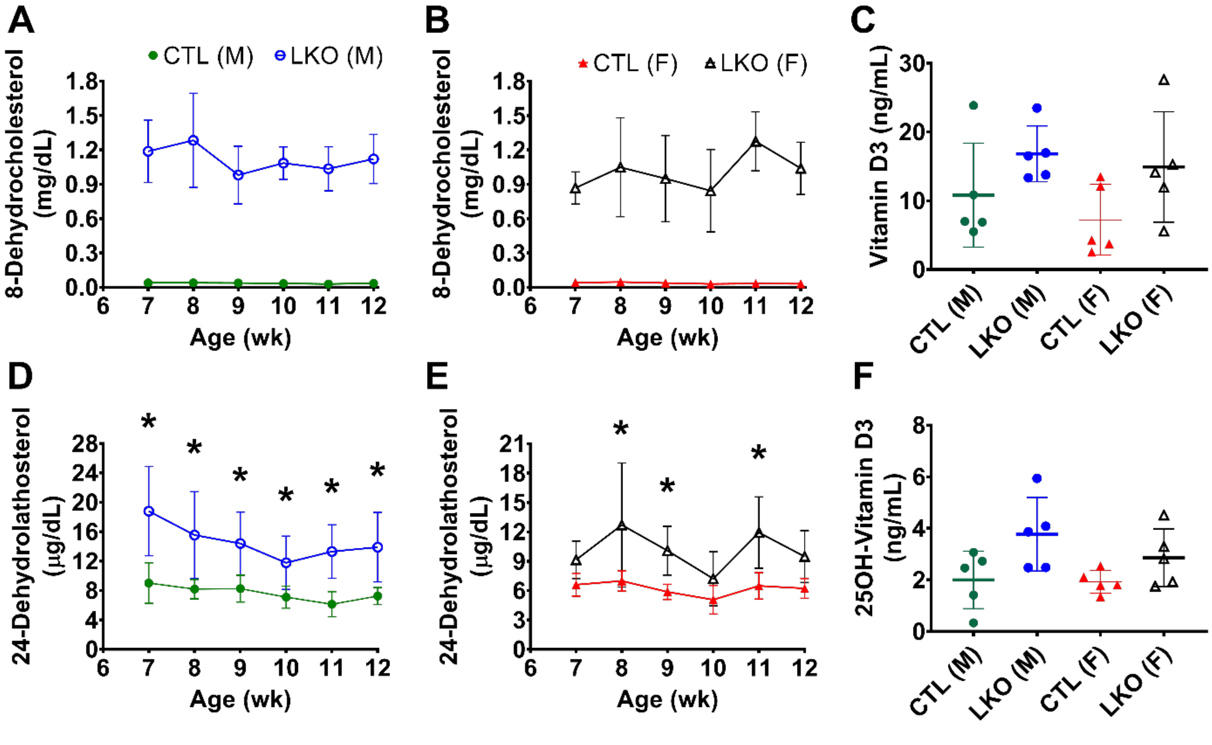


**Figure S6. Serial cholesterol precursor levels and vitamin D levels.** Weekly profiles of plasma 8-dehydrocholesterol (males: panel A, females: panel B) and 24-dehydrolathosterol (males: panel D, females: panel E), and terminal vitamin D3 and 25OH-vitamin D3 (panel C & F) are shown. Levels of both 8-dehydrocholesterol and 24-dehydrolathosterol (DHL) are elevated in LKO mice though the increase in DHL was not consistent across all time points. There were no significant differences detected in vitamin D3 or 25OH-vitamin D3 levels in plasma of LKO mice compared to control mice. Males are represented by circles, and females by triangles. LKO mice are represented by open symbols and CTL mice by closed symbols. Bars denote the mean ± 1SD, (*=p<0.05 vs sex-matched controls). N=5-6 per group.

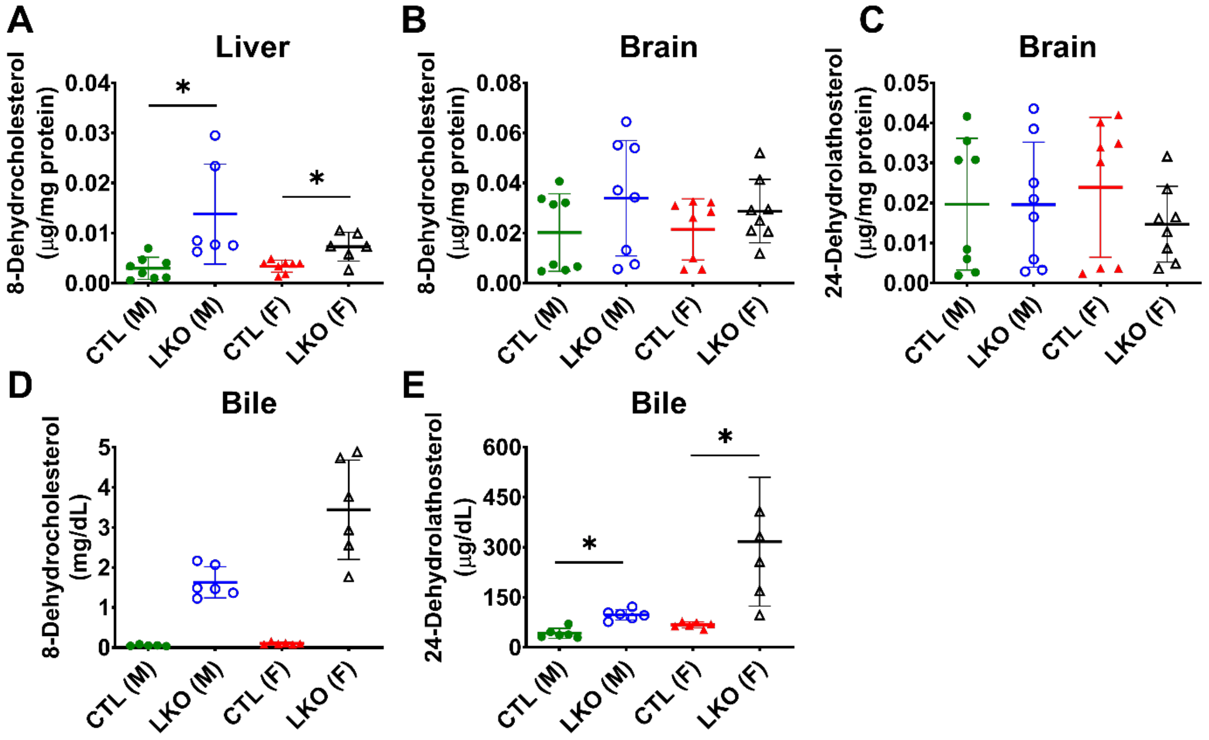


**Figure S7. Sterol precursor levels in liver, brain, and bile.** Levels of 8-dehydrocholesterol (8-DHC) in liver (panel A) brain (panel B) and bile (panel D) and levels of 24-dehydrolathosterol (DHL) in brain (panel C) and bile (panel E) of mice with liver-specific knockout (LKO) compared to sex-matched controls (CTL) are shown. As expected, 8-DHC levels were elevated in LKO livers. Brain samples showed no differences in 8-DHC or DHL levels between LKO and CTL mice. Biliary 8-DHC is undetectable in control mice while both 8-DHC and DHL were very elevated in bile of LKO mice. Males are represented by circles, and females by triangles. LKO mice are represented by open symbols and CTL mice by closed symbols. Bars denote the mean ± 1SD, (*=p<0.05 vs sex-matched controls). N=6-8 per group for liver, N=8 per group for brain, and N=5-6 per group for bile measurements.

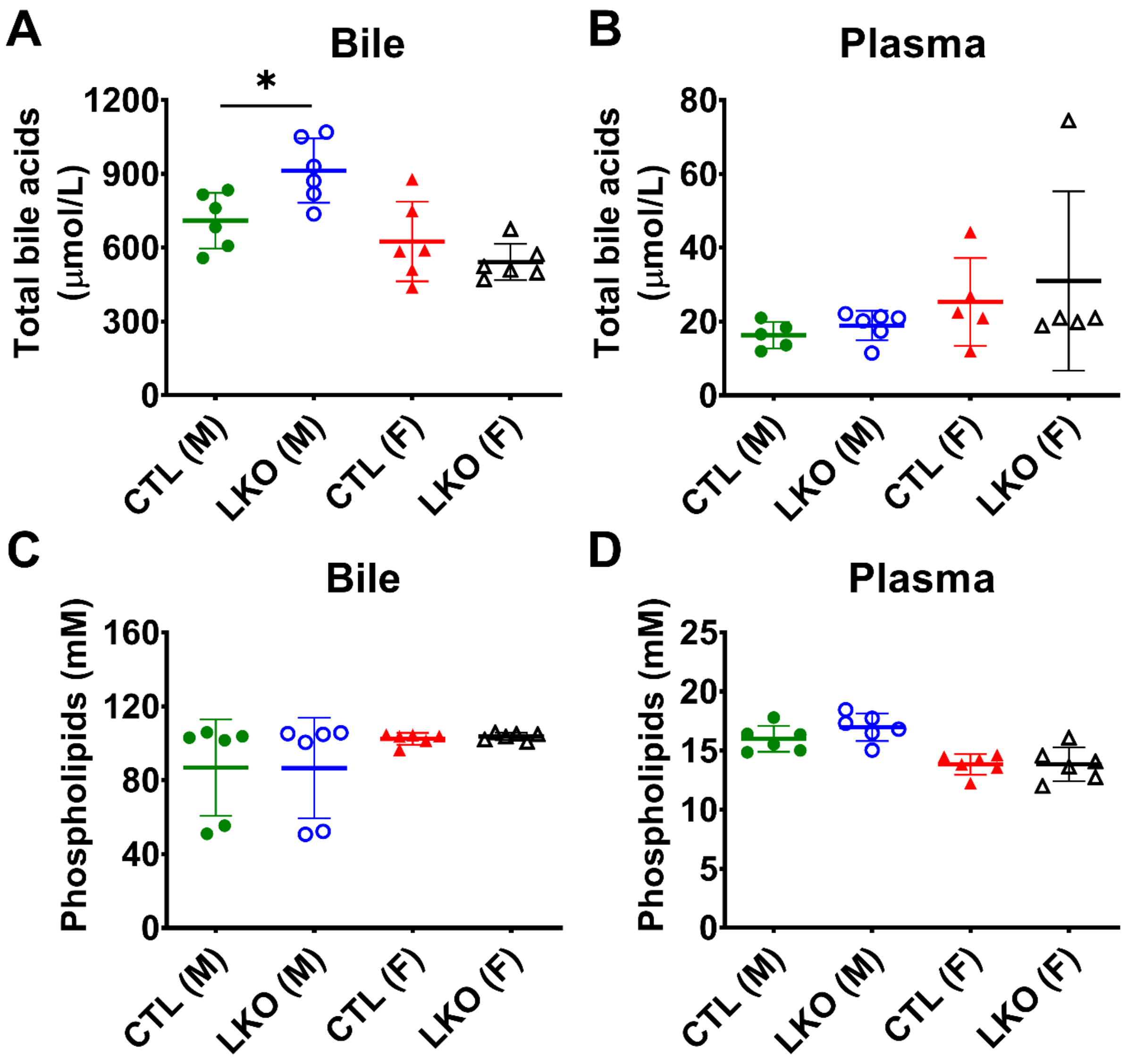


**Figure S8. Biliary and plasma bile acid and phospholipid levels.** Although male LKO mice showed statistically increased total bile acid levels in bile (panel A), biliary phospholipid levels (panel C), and plasma total bile acid (panel B) and phospholipid levels (panel D) were unchanged between LKO and control mice. N=5-6 per group. Males are represented by circles, and females by triangles. LKO mice are represented by open symbols and CTL mice by closed symbols. Bars denote the mean ± 1SD, (*=$p<0.05$ vs sex-matched controls).

**Table S1: Different primers used for validating LKO and CTL mice**

| Primer | Forward | Reverse | Product size (bp) |
|---|---|---|---|
| **For tail snip genotyping** | | | |
| Flox | TGGCTGTGTAGTCTTC AGGTGCT | TCAGCCTAAGACGGT GTGAAAAC | 496 bp (Flox) 374 bp (WT) |
| Cre | GCATTACCGGTCGATG CAACGAGTGATGAG | GAGTGAACGAACCTG GTCGAAATCAGTGCG | 408 bp |
| **For RNA exon splicing** | | | |
| Exon 5-9 | TGCTTTATTCCTGGCTT CC (Exon 5) | TGGTTGGTCATTCGG AAG (Exon 9) | 742 (WT) 610 (KO) |
| Exon 6-9 | TGGTTTGTGAACGCTT ACC (Exon 6) | TGGTTGGTCATTCGG AAG (Exon 9) | 596 (WT) 464 (KO) |
| Exon 7-9 | GCTGTTCTTCAATGGA CGAC (Exon 7) | TGGTTGGTCATTCGG AAG (Exon 9) | 360 (WT) 228 (KO) |
| **For genomic validation of exon 8** | | | |
| Exon 8 | CCCTAGTCACAACTTA TGGCCCTTG | TCAGCCTAAGACGGT GTG AAAAC | 1188bp (WT) 259bp (KO) |
